# Micro-CT Study of *Rhynchonkos stovalli* (Lepospondyli, Recumbirostra), with Description of Two New Genera

**DOI:** 10.1371/journal.pone.0127307

**Published:** 2015-06-10

**Authors:** Matt Szostakiwskyj, Jason D. Pardo, Jason S. Anderson

**Affiliations:** 1 Department of Biological Sciences, University of Calgary, Calgary, Alberta, Canada; 2 Department of Comparative Biology and Experimental Medicine, University of Calgary, Calgary, Alberta, Canada; Team 'Evo-Devo of Vertebrate Dentition', FRANCE

## Abstract

The Early Permian recumbirostran lepospondyl *Rhynchonkos stovalli* has been identified as a possible close relative of caecilians due to general similarities in skull shape as well as similar robustness of the braincase, a hypothesis that implies the polyphyly of extant lissamphibians. In order to better assess this phylogenetic hypothesis, we studied the morphology of the holotype and three specimens previously attributed to *R*. *stovalli*. With the use of micro-computed x-ray tomography (μCT) we are able to completely describe the external and internal cranial morphology of these specimens, dramatically revising our knowledge of *R*. *stovalli* and recognizing two new taxa, *Aletrimyti gaskillae* gen et sp. n. and *Dvellacanus carrolli* gen et sp. n. The braincases of *R*. *stovalli*, *A*. *gaskillae*, and *D*. *carrolli* are described in detail, demonstrating detailed braincase morphology and new information on the recumbirostran supraoccipital bone. All three taxa show fossorial adaptations in the braincase, sutural articulations of skull roof bones, and in the lower jaw, but variation in cranial morphology between these three taxa may reflect different modes of head-first burrowing behaviors and capabilities. We revisit the homology of the supraoccipital, median anterior bone, and temporal bone of recumbirostrans, and discuss implications of alternate interpretations of the homology of these elements. Finally, we evaluate the characteristics previously used to unite *Rhynchonkos stovalli* with caecilians in light of these new data. These proposed similarities are more ambiguous than previous descriptions suggest, and result from the composite nature of previous descriptions, ambiguities in external morphology, and functional convergence between recumbirostrans and caecilians for head-first burrowing.

## Introduction

Determining the origin of lissamphibians (frogs, salamanders, and caecilians) has been a controversial problem in tetrapod phylogeny [1]. Recent discoveries have confidently placed batrachians (frogs and salamanders) within dissorophoids temnospondyls on the basis of numerous synapomorphies [2,3] but firm answers on the problem of caecilian origins have remained elusive. This is due in part to the very poor early fossil record of caecilians, which is restricted to two taxa: *Eocaecilia micropodia* from the Early Jurassic of Arizona [4–6] and the more fragmentary *Rubricaecilia monbaroni* from the Early Cretaceous of North Africa [7]. These taxa are separated by more than 40 million years, and a larger gap exists between *E*. *micropodia* and possible Paleozoic-aged sister taxa. These substantial gaps in the fossil record coincide with a similar gap in morphology; although braincase morphology of *E*. *micropodia* established its identity as a stem-caecilian with certainty [6], the morphology of the skull, braincase, and postcranium of *E*. *micropodia* is specialized enough to have no comparison among Paleozoic tetrapods. Several hypotheses of caecilian ancestry have been forwarded. A first hypothesizes a polyphyletic Lissamphibia, with caecilians originating within either recumbirostran [2,8–11] or lysorophian [12–14] lepospondyls, and are thus more closely related to amniotes than other lissamphibian orders. Another, more widely accepted, hypothesizes lissamphibian monophyly within dissorophoid temnospondyls [3,6,15]. Strong morphological evidence for each of these hypotheses has been difficult to find, and in some cases (e.g. [6,15]) may reflect soft-tissue support for a monophyletic Lissamphibia rather than clear similarities between caecilians and putative Paleozoic relatives.

Recumbirostra, a group of ‘microsaur’ ‘lepospondyls’ for which monophyly has been reasonably established, are one group which has received significant consideration as possible caecilian relatives [2, 8–11]. Recumbirostrans are characterized by the presence of a shovel-like ‘recumbent’ snout and a strap-like atlantoccipital articulation, as well as a bullet-shaped skull and heavily-reinforced stegokrotaphic skull roof, and are diverse but rare components of Carboniferous-Permian terrestrial vertebrate communities. Similar morphology has been noted between the braincases of the recumbirostran ‘microsaur’ *Rhynchonkos stovalli* and caecilians, specifically in the expansion of ossifications within the antotic region (so-called ‘pleurosphenoids’) and of the anterior braincase (‘sphenethmoids’), with additional similarities including: parallel rows of teeth in the palate and lower jaw, passage of the metotic foramen through the exoccipital, elongate postcranium, and a robust, bullet-like skull [8, 9]. Recent work on the brachystelechid recumbirostran *Carrolla craddocki* [16] has confirmed the presence of a caecilian-like consolidation of the otoccipital region of the braincase into an ‘*os basale*’ as well as demonstrated the presence of a more massive ossification of the anterior braincase than previously thought, but they attributed this similarity to convergence driven by head first burrowing. Similar work on the ostodolepid *Nannaroter mckinziei* [17] and the generalized recumbirostran *Huskerpeton englehorni* [18] has confirmed the existence of similar consolidation of cranial ossification throughout the Recumbirostra, but with the caveat that the full suite of caecilian-like characteristics described in *Rhynchonkos stovalli* are not seen in any of these taxa. The presence of such a suite of morphology in *Rhynchonkos stovalli* itself is based on observations of numerous specimens with nonoverlapping representation of skeletal elements [8, 9, 19]. Caecilian-like gross skull morphology was drawn from two skulls (FMNH UR-1039 and FMNH UR-1040) that, while roughly similar overall, show differences in the overall shape of the skull as well as the proportions of specific bones of the dermal skull. Caecilian-like morphology of the lateral wall of the braincase and presence of a lissamphibian-like opercular bone was interpreted from a third specimen (UCMP 202940) with dermal skull elements not directly comparable to either complete skull. Caecilian-like trunk elongation was inferred from a fourth specimen (UCMP 202927) with a crushed skull with unclear affinities to any of the three specimens represented by well-preserved skulls. A second row of teeth in the lower jaw was inferred from a fragmentary lower jaw not associated with any material assigned to this taxon. Phylogenetic analyses of early tetrapods and the early caecilian *Eocaecilia micropoda* continue to recover a close relationship between *R*. *stovalli* and caecilians [2,10,11,18] but these results rely heavily on the assumption that the caecilian-like suite of morphology attributed to *Rhynchonkos* does not represent an amalgam of caecilian-like characteristics drawn from several distinct taxa into an artificially caecilian-like form. Moreover, recent analyses incorporating a more thorough sample of caecilian morphology have rejected a close relationship between caecilians and recumbirostrans in favor of a monophyletic Lissamphibia within temnospondyls [6,15].

We recently had the opportunity to study the material used by Carroll and Gaskill [19] to prepare their description of the genus. These specimens were CT scanned in our facility at the University of Calgary to expand our comparative database of the internal morphology of recumbirostrans and to better understand patterns of evolution in this important anatomical and developmental module. The variation in cranial shape illustrated by Carroll and Gaskill [19] is consistent with the variation observed in the specimens themselves, but is not easily attributable to either post-mortem deformation or intraspecific variation. Similarly, the internal braincase anatomy differs strikingly between morphotypes. These differences confirm that the reconstruction, description, and systematic paleontology of Carroll and Gaskill [19] is based on a composite of at least three distinct taxa; the general description of the skull was combined from two distinct taxa, the morphology of the braincase drawn from a third, and morphology of the lower jaw augmented from observations of an isolated jaw distinct from all three skull morphotypes. On the basis of these observations, we here redescribe *Rhynchonkos stovalli* and erect two new species of recumbirostran previously assigned to *R*. *stovalli*.

## Materials and Methods

### Specimens studied

The type specimens of *Rhynchonkos stovalli* (FM-UR 1039) and *Aletrimyti gaskillae* (FM-UR 1040) are permanently reposited in the vertebrate paleontology collections of the Field Museum in Chicago, Illinois, USA. The type specimen of *Dvellacanus carrolli* (UCMP 202940) and a specimen attributed to *Aletrimyti gaskillae* (UCMP 202927) are permanently reposited in the vertebrate paleontology collections of the University of California Museum of Paleontology in Berkeley, California, USA. No permits were required for the described study, which complied with all relevant regulations.

### MicroCT

All specimens underwent high-resolution x-ray computed tomography (HRXCT) at the Anderson lab (Foothills Campus, University of Calgary, Calgary, Canada) in a SkyScan 1173 micro-CT. The resulting data files were cropped using ImageJ 1.46r [20]. All empty images were removed, and the stack was down-sampled to reduce computational strain.

The data sets then were imported into Amira 5.4 (VGS, Burlington, MA, USA) as a series of stacked image files. Initial surface models were generated using the Volume Render module. Individual elements were volumized with the Label Field module at varying threshold levels. These different threshold values were chosen to maximize the contrast between the bony elements and the background. The scans were visualized by first down-sampling the data sets using the Resample module, then the 3D images were generated using the Surface Generation and Surface View modules. Measurements were calculated using the Measurement module. Each scan was performed at a different resolution and power level, as reported below.

The holotype skull of *Rhynchonkos stovalli* (FM-UR 1039) was scanned on October 2, 2013 at a voltage of 60 kV and a current of 0.090 mA. The resulting 950 images had a resolution of 1120 px by 1120 px, 16-bit gray scale, with a pixel size of 23.43 μm. The scan data were then down-sampled to 623 images at 539 px by 350 px, 8-bit gray scale.

The holotype skull of *Aletrimyti gaskillae*, *gen*. *et sp*. *nov*. (FM-UR 1040) was scanned on May 8, 2013 at a voltage of 100 kV and a current of 0.062 mA. The resulting 909 images in the transverse plane had a resolution of 1120 px by 1120 px, 16-bit gray scale, with a pixel size of 22.01 μm. The scan data was then down-sampled to 455 images at 699 px by 436 px, 8-bit gray scale.

The skull of UCMP 202927, attributed to *Aletrimyti gaskillae*, *gen*. *et sp*. *nov*., was scanned on May 8, 2013 at a voltage of 100 kV and a current of 0.062 mA. The resulting 950 images in the transverse plane had a resolution of 1120 px by 1120 px, 16-bit gray scale, with a pixel size of 25.56 μm. The scan data were then down-sampled to 705 px by 323 px, 8-bit gray scale.

The holotype skull of *Dvellecanus carrolli*, *gen*. *et sp*. *nov*. (UCMP 202940) was scanned on September 24, 2013 at a voltage of 77 kV and a current of 0.084 mA. The resulting 665 images in the sagittal plane had a resolution of 1120 px by 1120 px, 16-bit gray scale, with a pixel size of 21.30 μm. The scan data were then down-sampled to 181 images at 539 px by 350 px, 8-bit gray scale.

### Nomenclature Acts

The electronic edition of this article conforms to the requirements of the amended International Code of Zoological Nomenclature, and hence the new names contained herein are available under that Code from the electronic edition of this article. This published work and the nomenclatural acts it contains have been registered in ZooBank, the online registration system for the ICZN. The ZooBank LSIDs (Life Science Identifiers) can be resolved and the associated information viewed through any standard web browser by appending the LSID to the prefix 2E16-4672-9068-2FD8DCD46200. The electronic edition of this work was published in a journal with an ISSN, and has been archived and is available from the following digital repositories: PubMed Central, LOCKSS.

## Results

### Systematic Paleontology

Lepospondyli Zittel 1888

Recumbirostra Anderson 2007

*Rhynchonkos* Schultze & Foreman 1981

***Rhynchonkos stovalli*** Olson 1970

*Goniorhynchus stovalli* Olson 1970

#### Holotype

FM-UR 1039, complete articulated skull from Fairmont Shale (Hennessey Group) outside Norman, Oklahoma, USA.

#### Locality and horizon

The Norman locality has previously been reported as preserving fossils in Bed 5 within the lower 20 meters of the Hennesey Formation [21] within the Annadarko Basin of Oklahoma. The Hennesey has since been subdivided into the Fairmont Shale, Kingmont Siltstone, and Salt Plains Formation [22,23]. The Norman locality exposes rocks of the Fairmont Shale (Hennessey Group), in a hillside southeast of Norman, Cleveland County, Oklahoma [21,24]. The Fairmont Shale is considered equivalent to the Choza Formation (Clear Fork Group) of Texas [21] and is therefore Kungurian (280–270.6 Ma) in age.

#### Revised Diagnosis

Small recumbirostran with the following combination of features: median buttress of premaxillae present, with broad shelf-like articulation with the overlying nasal pair; occipital surface nearly straight in dorsal view; cheek not emarginated between squamosal and jugal; posterolateral process of frontals present; parietal pair wider than frontal pair; skull roof contribution of postparietals anteroposteriorly abbreviated; postorbital small; maxilla extends to posterior margin of orbit; maxilla participates extensively in ventral margin of orbit; coronoid eminence twice as deep as mandibular ramus of jaw; coronoid not laterally exposed; depth of surangular greater than 50% its length; ossification of columella ethmoidalis not reaching skull roof; pleurosphenoids not in contact with sphenethmoids; cultriform process of parasphenoid does not reach anterior margin of sphenethmoids.

### *Aletrimyti gaskillae* gen. et sp. nov.

urn:lsid:zoobank.org:act:D3E4E1BC-2A2F-4CEC-ABD2-C9A04B11994C

*Goniorhynchus stovalli* Olson 1970

*Rhynchonkos stovalli*, Schultze & Foreman 1981

#### Etymology

The genus name is derived from the Greek αλέτρι (‘aletri’ = plow) and μύτη (‘myti’ = nose). ‘Gaskill’s plow snout’, in honor of Pamela Gaskill for her work in preparing numerous microsaur specimens for the RM collections, and for her excellent illustrations of many lepospondyl taxa.

#### Holotype

FM-UR 1040, complete skull and lower jaws.

#### Referred Specimen

UCMP 202927 (previously UCLA VP-2927), partially crushed skull

#### Horizon and Locality

Same as for *Rhynchonkos stovalli*, above.

#### Diagnosis

Small recumbirostran with the following combination of characters: median anterior braincase bone with well-developed Y-shaped ossification of columella ethmoidalis bracing against skull roof; strongly pointed snout with strongly recumbent premaxilla flooring nearly the entire length of the external naris; lacrimal deeply waisted between orbit and external naris; maxilla does not reach posterior margin of the orbit; ventral orbital process of lacrimal reaches suborbital process of jugal; broad suture between palatine process of prefrontal and palatine;skull diamond-shaped in dorsal view; postparietals converge to a point posteriorly; occipital contribution of postparietals greater than 60% their size; parietal pair no wider than frontal pair; cheek deeply emarginated, with contact between jugal and squamosal reduced or absent; quadrate anteriorly-displaced greater than 30% the length of the skull; coronoid eminence no more than 130% depth of the mandibular ramus; anterior process of coronoid extends more than 50% of the tooth row; depth of surangular less than 50% its length; pleurosphenoids form sutural contact with posterior margin of sphenethmoids.

### *Dvellecanus carrolli*, gen. et sp. nov.

urn:lsid:zoobank.org:act:00681BA1-8D77-47D8-8C91-83A4CAF098EB*Goniorhynchus stovalli* Olson 1970

*Rhynchonkos stovalli* Schultze & Foreman 1981

#### Etymology

The genus name is based on an anagram for “Cleveland”, the Oklahoma county in which the type locality was discovered. The species name honors Robert L. Carroll, whose monographic work,“The Order Microsauria” [19], is a landmark work in microsaur morphology and systematics.

#### Holotype

UCMP 202940 (previously UCLA-VP 2940), partial skull with right cheek removed to expose braincase.

#### Horizon and Locality

Same as for *Rhynchonkos stovalli*, above.

#### Diagnosis

Small recumbirostran with the following combination of characters: exoccipitals and basioccipital fused; snout strongly downturned, with premaxilla-nasal suture displaced ventral to cultriform process of parasphenoid; coronoid process exposed in lateral view; anterior process of coronoid short; nasal pair wider than frontal pair; snout rounded in dorsal view; prefrontal approaching external naris; dorsal lamina of lacrimal level with dorsal margin of orbit; ventral process of lacrimal approaches suborbital process of jugal; postorbital large; transverse flange of pterygoid entirely concave; retroarticular process absent; median anterior braincase bone absent; sphenethmoids with short ventral suture to pleurosphenoids; lateral ascending processes of supraoccipital short and squared-off.

### Description of *Rhynchonkos stovalli*

#### General

**Fig 1:**
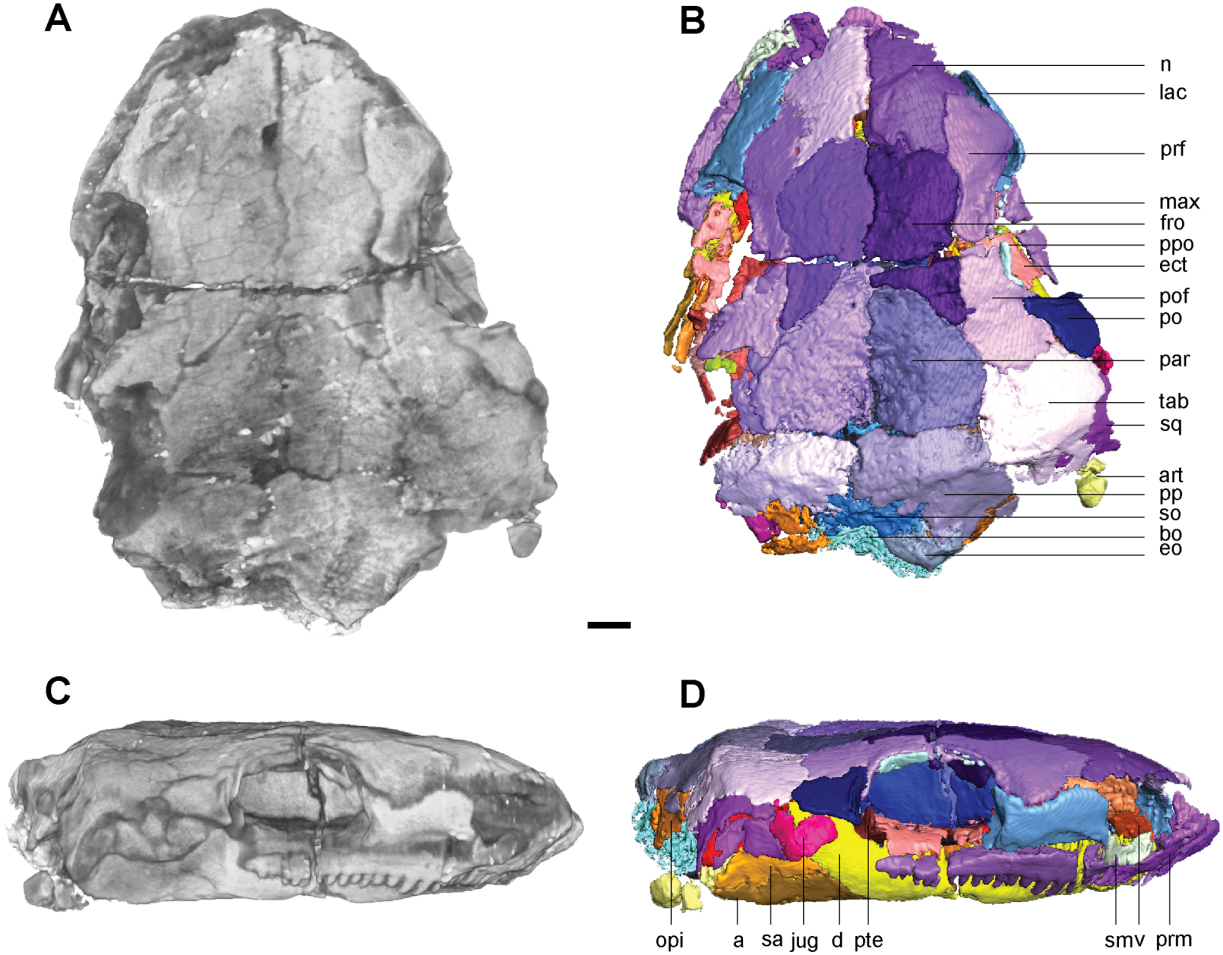
Volume renderings and volumizations of *Rhynchonkos stovalli* (FM-UR 1039). Shown in dorsal view (**A**, **B**) and right lateral view (**C**, **D**). Volume renderings (**A**, **C**) depict the specimen after being submitted to HRXCT. Volumizations (**B**, **D**) show the bony elements after they have been isolated. Scale bar equals 1mm. Abbreviations: **a**, angular; **art**, articular; **bo**, basioccipital; **d**, dentary; **ect**, ectopterygoid; **eo**, exoccipital; **fro**, frontal; **jug**, jugal; **lac**, lacrimal; **max**, maxilla; **n**, nasal; **opi**, opisthotic; **par**, parietal; **po**, postorbital; **pof**, postfrontal; **pp**, postparietal; **ppo**, palpebral ossification; **prf**, prefrontal; **prm**, premaxilla; **pte**, pterygoid; **sa**, surangular; **sm**, septomaxilla; **so**, supraoccipital; **sq**, squamosal; **tab**, tabular; **v**, vomer.

The skull of FM-UR 1039 is slightly deformed (Fig 1A and 1C). The maxilla and premaxilla have been separated from the skull roof and shifted laterally. The postorbital, tabular, squamosal, and jugal are all missing from the left side, and only a fragment of the jugal is present on the right. There is a transverse crack in the skull that runs through the midline of the orbits, separating the pre- and postfrontals. The left ectopterygoid has been shifted dorsally and is visible within the orbit.

In dorsal view the skull is roughly egg-shaped, broadest at the posterior of the parietals, with a bluntly rounded snout, and rounded off at the occipitals (Fig 1A and 1B). The nasals are shorter than the frontals and parietals, which are of equal length, and the parietals are wider than the nasals and frontals, which are of equal width. The bone is smooth, with no evidence of sculpture or ornamentation. Due to a left-lateral shifting of the jaw elements the left orbit has been re-oriented, rendering it obscured from lateral view and only visible in dorsal view. Seen in lateral view the skull is long and flat with rounded ends (Fig 1C and 1D). The rounded snout is slightly downturned, although this is probably a taphonomic artifact as the premaxillae no longer articulate with the nasals. A reconstruction of the premaxillae and nasals is consistent with morphology described as ‘shovel-headed’ in amphisbaenids [25]. The right orbit, unaffected by the lower jaw shift, is vertically oriented and circular. The external nares are completely distorted, and only the posterodorsal margin of the left one has been preserved. It is elliptical, with its long axis horizontally oriented and anterolaterally directed. The jaws are preserved in tight articulation with the coronoid process of the right dentary shifted dorsally and located medial to the postorbital. The posterior lower left jaw is not preserved.

#### Skull Roof

The premaxilla is tall and weakly recumbent (Fig 1B and 1D). It is roughly the same width as the nasal and half its length. The premaxilla is overlapped by the nasal, and no dorsal exposure is present. The premaxilla forms the anterior margin of the external naris, and the tooth row forms the anteromedial margin of the choana. A weak dorsoventral buttress on the dorsomedial margin of the premaxilla braces it against the nasal. A groove on the posterior portion of the premaxilla holds the septomaxilla. There are five large, peg-like teeth on the premaxilla.

The septomaxilla is a long, dorsally concave element that nearly spans the length of the naris. It contacts the lacrimal posteriorly and the premaxilla anteriorly, following the contours of both elements to border the external naris (Fig 1D).

The nasal is rectangular, with a small descending process anterior to the prefrontal that nearly reaches the lacrimal, contributing to the anterodorsal border of the external naris (Fig 1B and 1D). A straight ridge at the anteromedial margin, laterally-oriented and roughly half the width of the nasal, overlaps the premaxilla and forms the dorsal border to the external naris.

**Fig 2:**
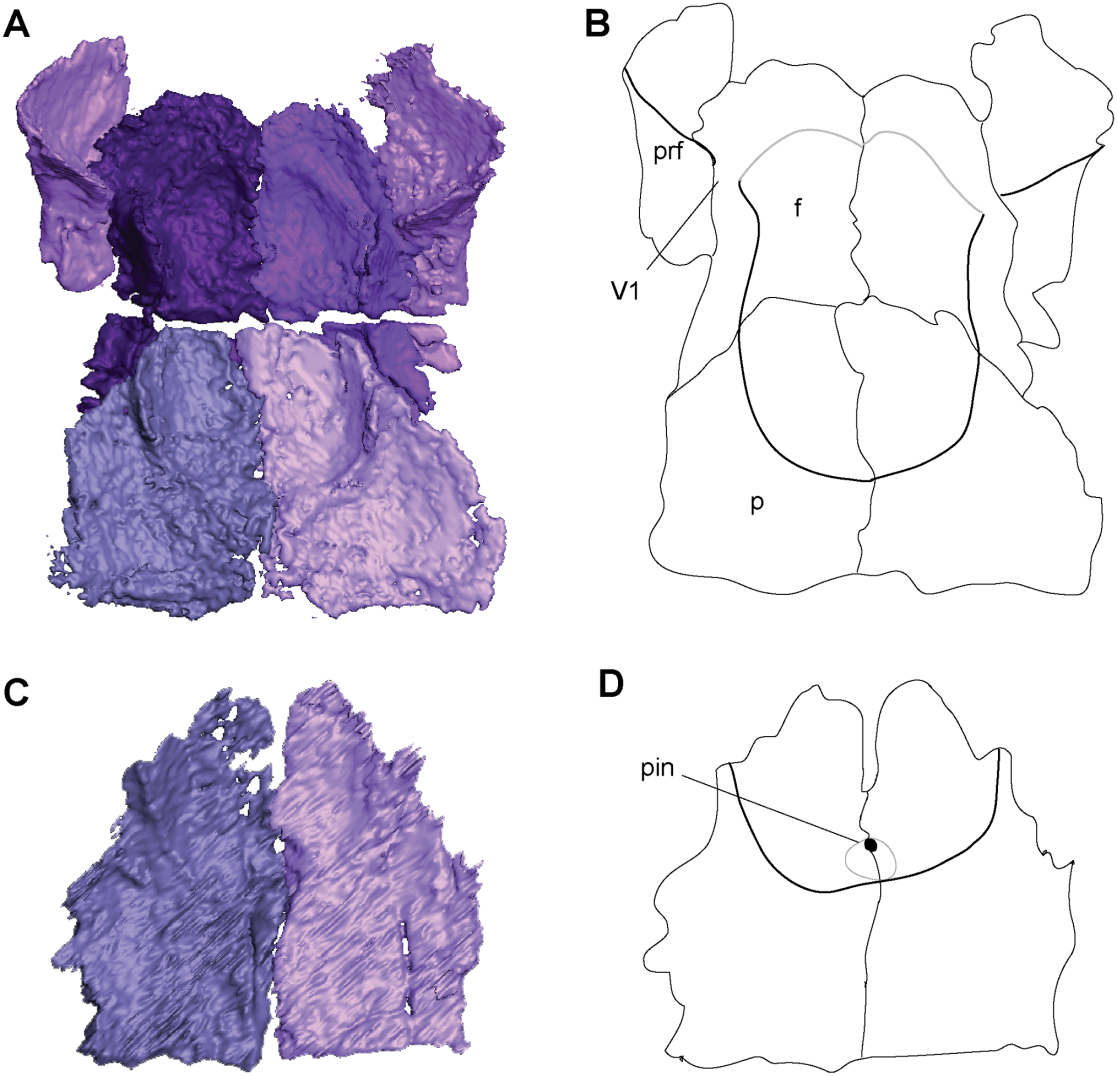
Ventral skull roof reconstructions and interpretive drawings of the descending flange of the prefrontals, frontals, and parietals. *Rhynchonkos stovalli* (FM-UR 1039); (**A**, **B**) and *Aletrimyti gaskillae*, *gen*. *et sp*. *nov*. (FM-UR 1040); (**C**, **D**), in ventral view, based on select micro-CT images. Bold line indicates the descending flange of the prefrontal, frontal, and parietal. Grey line indicates a depression in the bone. Not shown to scale. Abbreviations: **f**, frontal; **p**, parietal; **pin**, pineal foramen; **prf**, prefrontal; **V1**, foramen for the ophthalmic branch of the trigeminal nerve.

The frontal is large and trapezoidal, with its narrow posterior end oriented posterolaterally, and its broad anterior end borders the nasal. An anterior bulging of the frontal causes it to slightly overlap the prefrontal, tapering to a point posterolaterally and overlapping the postfrontal posterolaterally and the parietal posteriorly (Fig 1B). A descending internal flange runs along the ventral margin of the frontal (Fig 2A and 2B). This flange begins near the anterior orbital wall and spans the length of the frontal to nearly reach the parietal. It descends ventrally to approximately one-third the height of the orbit, where it overlaps an embayment for the length of the orbit in the sphenethmoid. Anteriorly the flange is separated from the anterior orbital wall by a small notch between the two, which is interpreted as the deep ophthalmic nerve foramen.

The parietal is a long triangular bone, as seen in dorsal view, which is overlapped by the frontal at its anterolateral edge, forming a scarf joint and giving the parietal an anteriorly tapered appearance (Fig 1B). A lateral flange on the parietal is overlapped by the postfrontal and tabular. The posterior edge of the parietal is straight where it abuts the postparietal. No pineal foramen is present. Internally a descending flange spans longitudinally from the anterior edge to slightly past the midpoint of the parietal, continuing that of the frontal flange, a small gap separating the two (Fig 2A and 2B). A small notch in the sphenethmoid, at an approximate level with the posterior orbital wall, accepts a lobe of the parietal flange. Posterior to this, the paired flanges meet at the midpoint (Fig 2C and 2D).

A depression on the underside of the frontals and parietals is visible, bordered by the descending flanges (Fig 2A and 2B). This depression is somewhat elliptical in shape: its posterior end is rounded and its anterior end is a double arch. Midway through the orbit, the flanges taper slightly inwards, giving the depression a slight pinching.

The postparietal is large and round, with a well-defined nuchal ridge separating the flat skull table and the downturned occiput (Fig 1D). The anterior edge is straight where it borders the parietal, forming a butt joint. A notch on the posteromedial margin at the midline suture where the postparietals meet exposes the supraoccipital (Fig 1B). Laterally the postparietal is overlapped by the tabular. The downturned occipital lappets of the postparietal ventrally contact the exoccipitals and provide a broad surface for the insertion of the epaxial musculature.

As in other ‘microsaurs,’ a single bone is present in the temporal region, interpreted here as the tabular (see discussion for more information). It is round and slightly larger than the orbit, with a nuchal ridge similar to that of the postparietals that separates the flat skull table and the downturned occiput (Fig 1D). The tabular overlaps the postfrontal anteromedially, the postparietal posteromedially, and the squamosal laterally. The left tabular is missing, exposing the underlapping facets on the parietal and postparietal.

The squamosal is a triangular bone with its points oriented dorsally, anteriorly, and posteriorly. It is approximately half the size of the tabular. The dorsal half of the squamosal is hidden from view by the overlapping tabular (Fig 1B and 1D). Anteriorly, it is overlapped by the jugal. No otic notch is present.

#### Circumorbitals

A ridge of raised bone borders the margins of the orbits. The orbits are very large and circular, each occupying approximately 28% of the area of the skull (Fig 1D).

The lacrimal forms the anterior margin of the orbit. It is a broad, hourglass-shaped element that extends to the septomaxilla and naris anteriorly (Fig 1D). A large concave facet that accepts a flange from the maxilla spans over half the length of the lacrimal’s ventral margin. Anterodorsally the lacrimal accepts a small process from the nasal. The dorsal margin of the lacrimal is incised by the prefrontal. The lacrimal participates in the antorbital wall via a broad medial flange, articulating with the palatine medially and broadly overlapping the descending flange of the prefrontal. The antorbital wall is pierced by three foramina. Two are present along the orbital margin, interpreted here as the dorsal and ventral entrance to the nasolacrimal canal. The third pierces the antorbital wall medial to the orbital margin, and is interpreted to represent the entrance of the lacrimal canal. Medially the lacrimal is smooth and concave. No suborbital process of the lacrimal is present, and the lacrimal does not contact the jugal.

The prefrontal forms the anterodorsal margin of the orbit. The prefrontal is a broad, teardrop-shaped element that does not reach the external naris (Fig 1D). The prefrontal articulates with the nasal anterodorsally and dorsally in a butt joint. An interdigitating articulation between the pre- and postfrontals excludes the frontals from participating in the dorsal margin of the orbit (Fig 1B). A contribution to the antorbital wall via a prefrontal flange is greatly overlapped by that of the lacrimal. The antorbital wall approaches the lateral wall of the sphenethmoid region, but is separated from the sphenethmoid and descending flange of the prefrontal by a notch, interpreted as a passage for the ophthalmic branch of the trigeminal nerve. Medially, the prefrontal is smooth and concave. A large sinus is formed by the prefrontal, lacrimal, and premaxilla, interpreted as for the nasal capsule and vomeronasal organ.

The postfrontal forms the posterodorsal margin of the orbit. It reflects the prefrontal and is similar in shape, although the thin supraorbital process is much shorter. Dorsally the postfrontal borders the frontal and parietal, the latter of which is slightly overlapped. The anterior process forms a notch to receive the prefrontal at a slight interdigitation. Posteriorly, the postfrontal overlaps the tabular, and ventrally a notch accepts the postorbital, which slightly overlaps the postfrontal (Fig 1D).

The postorbital forms the posterior edge of the orbit. It is small, approximately the same size as the posterior half of the postfrontal (Fig 1D). Along its anterior margin is a raised edge bordering the orbit, more pronounced than any of the other orbital rim elements. The dorsal margin overlaps the postfrontal. Posteriorly the postorbital borders the tabular and the jugal ventrally.

The jugal forms the posteroventral margin of the orbit, slightly overlapping the maxilla. The jugal articulates at the anterior edge of the squamosal, and between the squamosal and postorbital (Fig 1D).

**Fig 3:**
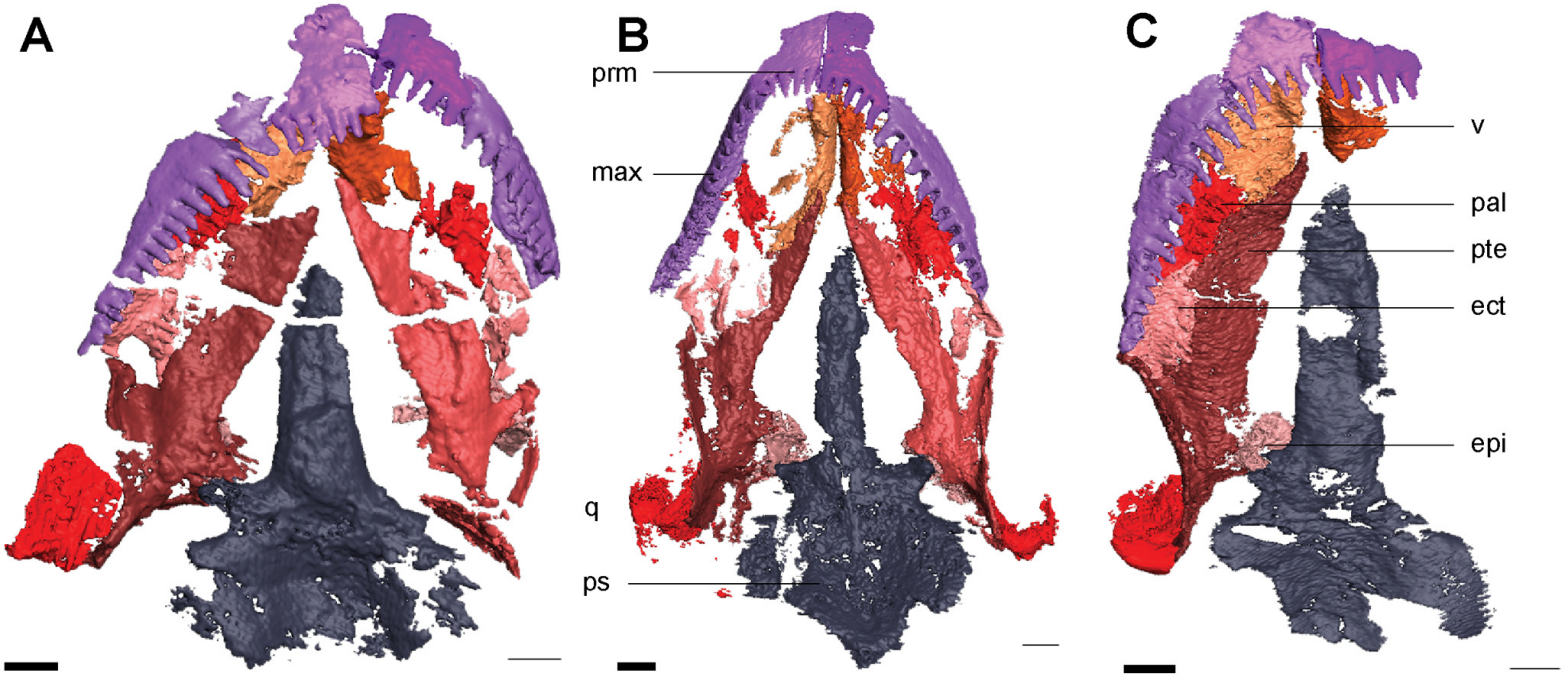
Isolated elements of the palate and neighboring elements in ventral view. **A**, *Rhynchonkos stovalli* (FM-UR 1039); **B**, *Aletrimyti gaskillae*, *gen*. *et sp*. *nov*. (FM-UR 1040); **C**, *Dvellecanus carrolli*, *gen*. *et sp*. *nov*. (UCMP 202940). Reconstructed from HRXCT images. Scale bar equals 1mm. Abbreviations: **ect**, ectopterygoid; **epi**, epipterygoid; **max**, maxilla; **pal**, palatine; **prm**, premaxilla; **ps**, parasphenoid; **pte**, pterygoid; **q**, quadrate; **v**, vomer.

The maxilla forms the ventral margin of the orbit. It is a long, slender element that extends from the posterior edge of the naris to the posterior margin of the orbit (Fig 1D). Approximately 15 non-pedicellate, peg-like, recurved teeth are on the maxilla. These teeth are of varying size: the first three are the largest, and they gradually decrease in size posteriorly. A small facet at the posterior end of the maxilla holds the jugal. Medially a shelf extends from the maxilla to meet the palatine and ectopterygoid, and continues anteriorly to form the lateral margin of the choana (Fig 3A). A small overlap joins the maxilla to the premaxilla in the region of the naris, with the maxilla excluded by the lacrimal.

A palpebral ossification is present in the dorsal margin of the right orbit. It is split in two by a large crack: the posterior half is large, while the anterior half is only present as small ossicles (Fig 1D). Presumably the palpebral was a single, large element that spanned the dorsal border of the orbit.

Whereas previous reconstructions have included a quadratojugal in *Rhynchonkos* [19], there is no evidence of one in the specimen.

#### Palate

The vomer is large and dorsally concave (Fig 3A). A process on the premaxilla articulates with the vomer anterolaterally, and these processes separate the ovoid choanae, which are located posteromedially to the external nares. A posterior process on the ventral margin of the vomer slightly overlaps the pterygoid. Posterolaterally the vomer is in firm contact with the palatine. The medial wall of the vomer is dorsoventrally-oriented, and the two vomers meet medially creating a septum along the midline of the braincase. The lateral wall of the vomer is splayed obliquely, and forms the posteromedial wall for the choana. Two conical teeth are preserved on a raised ridge that runs anteroposteriorly, medial to the choana, on the right vomer.

The palatine is a flat, square element roughly the same size as the vomer (Fig 3A). It is tightly sutured anteromedial to the vomer, with a small anterior concavity between the vomer and lateral maxilla that forms the posterior border to the choana. The medial edge of the palatine firmly contacts the pterygoid. A facet in the anterolateral margin of the palatine accommodates the medial edge of the maxilla. Posteriorly the palatine would have firmly contacted the ectopterygoid; however, both are preserved out of articulation. Three teeth on the left palatine and six on the right are continuous with those on the vomers and form a row parallel to the maxillary tooth row.

The ectopterygoid is a flat square, and similar in size to the palatine (Fig 3A). A small ridge along the lateral margin of the ectopterygoid accepts the overlapping maxilla, binding the two together tightly and leaving only a small posterolateral exposure between the maxilla and pterygoid. Approximately four teeth are preserved on the ectopteryoid at the posteromedial edge, in line with those on the vomer and palatine.

The pterygoid is the largest element of the palate, its length spanning from the midpoint of the maxilla to the midpoint of the basal plate of the parasphenoid (Fig 3A). The palatine process is long and anteriorly tapered, forming the lateral margin of the interpterygoid vacuity. The two processes are not preserved in articulation but likely met where a small facet in the vomer accepts the pterygoid. Toward the basipterygoid process the pterygoid slightly bows laterally. Posterior to it the quadrate ramus is tall and flat, approximately of a height with the quadrate. It is medially concave as it stretches from the basipterygoid process to midway through the basal plate of the parasphenoid. Where the quadrate ramus joins the palatine process the pterygoid is ventrally concave. The palatine process and transverse process form a U-shaped facet that accepts the ectopterygoid. The epipterygoids loosely contact the pterygoids, dorsal to the basipterygoid processes. No dentition is observed on the pterygoid.

**Fig 4:**
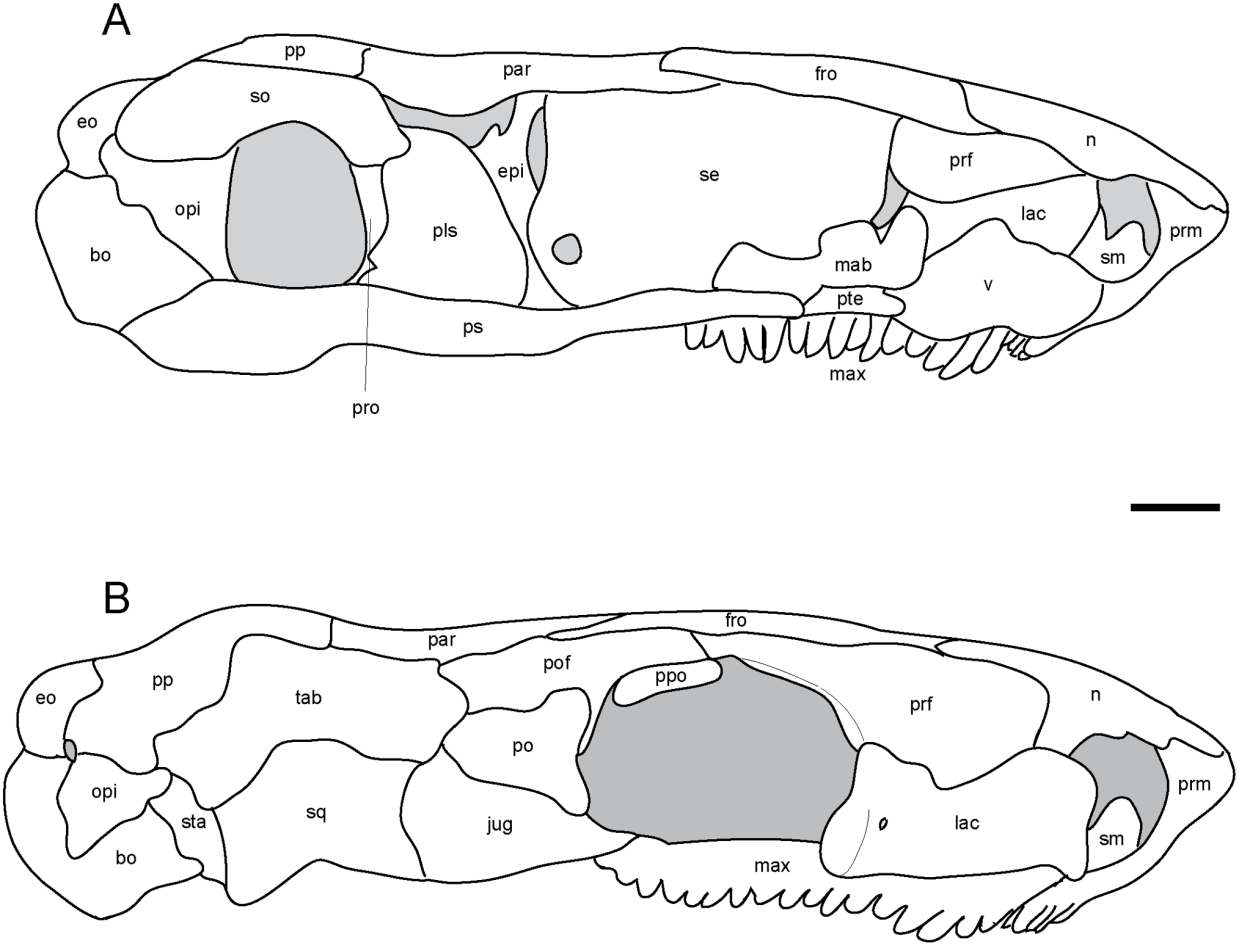
Interpretive drawings of *Rhynchonkos stovalli* (FM-UR 1039). **A**, medial view; **B**, right lateral view. Lower jaw has been removed to better reveal palate and cheek elements. Scale bar equals 1mm. Abbreviations: **bo**, basioccipital; **eo**, exoccipital; **epi**, epipterygoid; **fro**, frontal; **jug**, jugal; **lac**, lacrimal; **mab**, ‘median anterior braincase bone’; **max**, maxilla; **n**, nasal; **opi**, opisthotic; **par**, parietal; **pls**, pleurosphenoid; **po**, postorbital; **pof**, postfrontal; **pp**, postparietal; **ppo**, palpebral ossification; **prf**, prefrontal; **prm**, premaxilla; **pro**, prootic; **ps**, parasphenoid; **pte**, pterygoid; **se**, sphenethmoid; **sm**, septomaxilla; **so**, supraoccipital; **sq**, squamosal; **sta**, stapes; **tab**, tabular; **v**, vomer.

The epipterygoid is a dorsoventrally oriented flat sheet of bone that rests dorsal to the basipterygoid process, with a thin rod-like extension directed dorsomedially that reaches the skull roof (Fig 4A). It is bowed similar to the quadrate ramus of the pterygoid, which it presumably would have directly contacted. The posterolateral margin of the epipterygoid is narrowly separated from the quadrate (Fig 3A).

**Fig 5:**
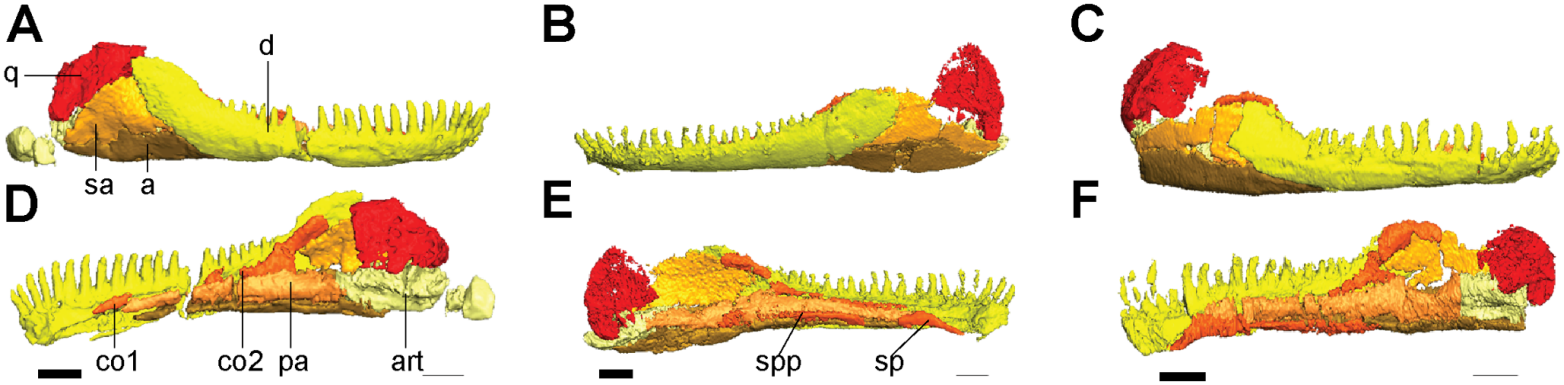
Isolated elements of the lower jaw and quadrate. Shown in right lateral (**A**, **C**), left lateral (**B**), and medial (**D**, **E**, **F**) views. **A**, **D**, *Rhynchonkos stovalli* (FM-UR 1039); **B**, **E**, *Aletrimyti gaskillae*, *gen*. *et sp*. *nov*. (FM-UR 1040); **C**, **F**, *Dvellecanus carrolli*, *gen*. *et sp*. *nov*. (UCLA-VP 2940). Reconstructed from HRXCT images. Scale bar equals 1mm. Abbreviations: **a**, angular; **art**, articular; **co1**, anterior coronoid; **co2**, posterior coronoid; **d**, dentary; **pa**, prearticular; **sa**, surangular; **sp**, splenial; **spp**, post-splenial.

The quadrate is large and robust. It is triangular in anterior view, with a point dorsally oriented. The trochlear ventral margin is preserved atop the articular (Fig 5D). The quadrate fails to reach the dorsal skull roof, but it contacts both the tabular and squamosal laterally. Medially the quadrate only slightly contacts the quadrate ramus of the pterygoid, and does not contact the epipterygoid. The stapes is separated from the quadrate by the quadrate ramus of the pterygoid, and there is no evidence of the two meeting.

#### Braincase and Occiput

**Fig 6:**
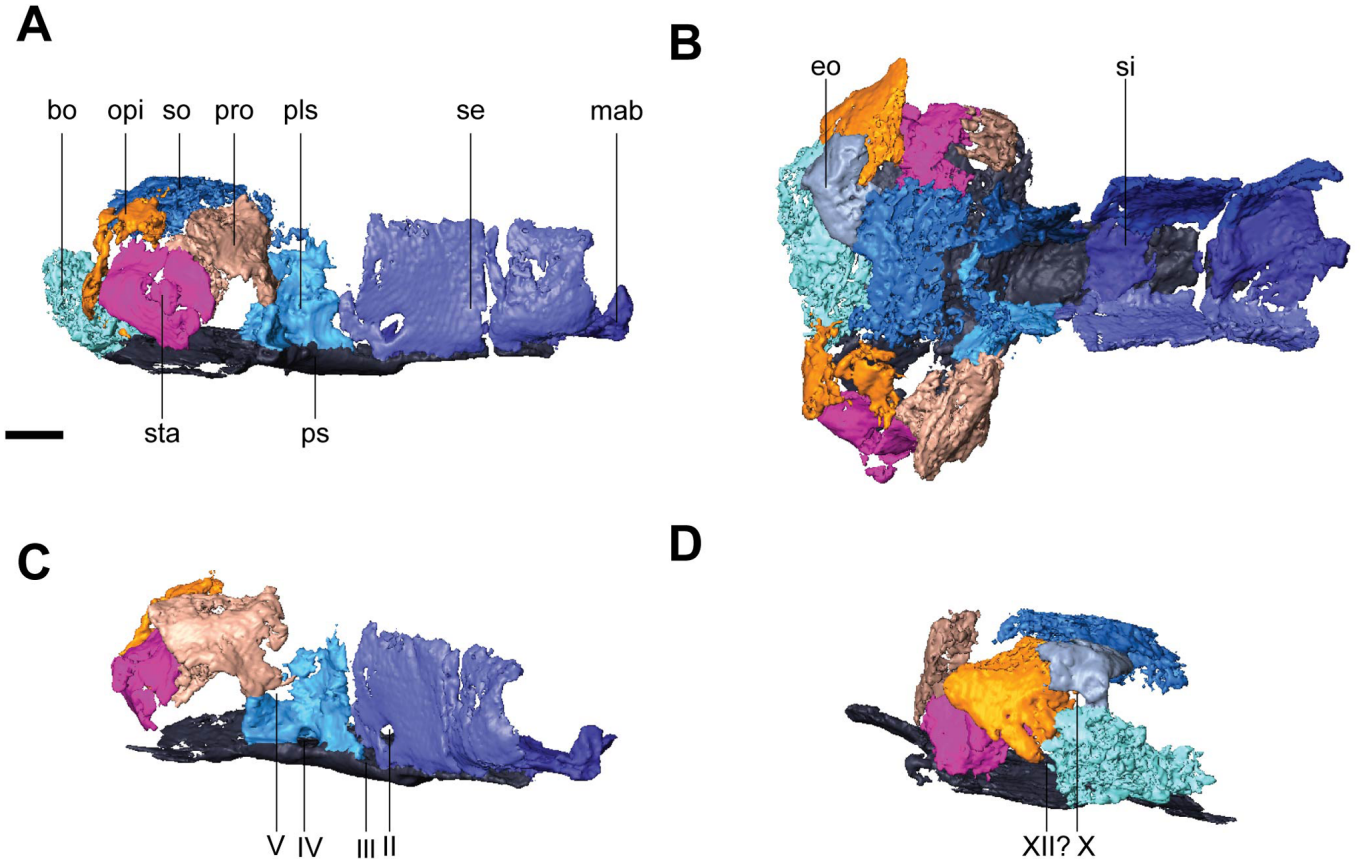
Isolated braincase elements of *Rhynchonkos stovalli* (FM-UR 1039). Shown in right lateral (**A**), dorsal (**B**), right anterolateral oblique (**C**), and left posterolateral oblique (**D**) views. Surrounding elements have been removed to expose braincase. Scale bars equal 1mm. Abbreviations: **bo**, basioccipital; **eo**, exoccipital; **mab**, ‘median anterior braincase bone’; **opi**, opisthotic; **pls**, pleurosphenoid; **pro**, prootic; **ps**, parasphenoid; **se**, sphenethmoid; **si**, ossification of the subiculum infundibulum; **so**, supraoccipital; **sta**, stapes; **II**, optic nerve foramen; **III**, oculomotor nerve foramen; **IV**, trochlear nerve foramen; **V**, fenestra prootica; **X**, foramen for the vagus nerve and jugular vein; **XII**, hypoglossal nerve foramen.

The braincase is robust, beginning at the anterior edge of the frontals and extending to the posterior edge of the parietals, dorsally meeting the dermatocranium (Fig 4A and Fig 6). It is rectangular in shape, maintaining its height and width throughout.

The parasphenoid forms the floor for the braincase. The cultriform process is long and wide, extending from the anterior margin of the orbit to midway through the occiput (Figs 3A and 6A). It is slightly concave dorsally, has a rounded anterior tip, and is at a level with the palatal elements. There is a slight anterior tapering, with the broadest section of the cultriform process where it joins the basal plate. At the posterior end of the cultriform process is a dorsal recess for the hypophyseal fossa, which is laterally walled by the pleurosphenoids (Fig 6B). Slightly posterior and lateral to the cultriform process are the basipterygoid processes. The basal plate is a broad rectangle that floors the otic capsules. Between the occiput and the otic capsules is a recess for the basicranial fenestra. A patch of denticles is preserved at the base of the cultriform process.

The sphenethmoids are tall and rectangular, flanking the cultriform process along its entire length, extending past it to the anterior margin of the orbit (Figs 4A and 6A). The sphenethmoids do not meet medially at any point along the braincase. The optic foramen is fully housed in the posteroventral corner of the sphenethmoid (Fig 6A and 6C). The posterior-most edge of the sphenethmoid provides the anterior half of the border for the oculomotor foramen (Fig 6A and 6C). The sphenethmoid reaches the dermatocranium dorsally. A descending flange from the frontal overlaps the sphenethmoid laterally, descending halfway through the sphenethmoid, and spanning approximately half the length of the orbit. The flange from the parietal, posterior to the level of the orbit, has a much smaller overlap than the frontal.

Medial to the sphenethmoids is an ossification of the subiculum infundibuli (Fig 6B). It is narrow, and connects the posterior ends of the sphenethmoids. It provides the anterior border to the hypophyseal fossa, and the posterior border to an unknown fossa located midway through the sphenethmoids.

The pleurosphenoids are posterior to the sphenethmoids. They are short, beginning at the midpoint of the hypophyseal fossa and extending to the basipterygoid processes. Here, a laterally-oriented process extends from the pleurosphenoid, posteriorly enclosing the basipterygoid process (Fig 6A–6C). A large foramen between this process and the prootic is interpreted as for the trigeminal nerve (Fig 6C). The pleurosphenoids fail to reach the dermatocranium. At the anteroventral end they contact the sphenethmoids tightly and contribute the posterior half of the border to the oculomotor nerve (Fig 6C). Dorsal to the basipterygoid processes is a foramen between the basal plate and the pleurosphenoids, interpreted as the trochlear foramen (Fig 6C). The dorsum sellae provides the posterior wall for the hypophyseal fossa, as well as the anterior wall for the basicranial fenestra. At their posterodorsal tip the pleurosphenoids contact the supraoccipital.

The sphenethmoids are connected anteromedially by an ossification in the region of the ethmoid (Fig 6A–6C), similar to the ‘median anterior braincase bone’ seen in *Carrolla* [16]. This ossification is wider than the cultriform process and half the length of the sphenethmoids. A short median process extends anterodorsally to the halfway point of the sphenethmoids.

The occiput is composed of a basioccipital, paired exoccipitals, and a supraoccipital (Fig 6A, 6B and 6D). The basioccipital is short, slightly wider than the base of the cultriform process, with a small overlap of the basal plate of the parasphenoid. It provides the floor for the foramen magnum. Only the short, knob-like right exoccipital has been preserved. Along with its missing counterpart, the exoccipitals would have flanked the basioccipital and provided the walls for the foramen magnum. The exoccipital is sutured to the postparietal dorsolaterally, the supraoccipital dorsomedially, and the basioccipital ventromedially. It is strongly underplated by the opisthotic.

Together, with the basioccipital, the exoccipitals create a flat posterior surface. Neither occipital condyle nor cotyle is preserved. A foramen is visible passing between the right exoccipital and the opisthotic, interpreted as the jugular foramen (Fig 6D). This is in contrast to the initial description of *Rhynchonkos* [19], where the jugular foramen is described as piercing the exoccipitals. A short supraoccipital articulates with the anteromedial margins of the exoccipitals and opisthotics. A pair of lateral processes, partially concealed by the postparietals, extends from the supraoccipital past the prootics to contact the ‘pleurosphenoids.’ It is short, with a pair of lateral processes that extend past the prootics to articulate with the pleurosphenoids. The supraoccipital is concave ventrally, roofing the foramen magnum.

#### Otic Capsules

The otic capsules are composed of a distinct anterior prootic and a posterior opisthotic, which are approximately of equal size (Fig 6A and 6B). They are stout elements that span the length of the relatively short basal plate of the parasphenoid, and are wide, approximately less than one-half of the basal plate’s width. The otic capsules fail to meet the dermatocranium anterodorsally, leaving a small sinus preserved between the dorsal margin of the prootic and the postparietal. Posterodorsally, the postparietal contacts the opisthotic where the postparietal is ventrally sloped. The otic capsule is arched where it encloses the fenestra vestibuli. A mediolaterally-oriented shelf extends on the dorsal margin of the otic capsules, reaching the supraoccipital. Although the otic capsules span the length of the basal plate, they only have small points of contact at the anterior and posterior margins of the basal plate. Posteriorly the otic capsules suture to the basioccipital and the exoccipitals. In dorsal view they are medially straight and laterally concave, bulging slightly past the lateral margins of the basal plate. Ventral to the dorsal shelf the margins for the horizontal semicircular canal are visible, evenly housed by both elements of the otic capsules. The lagenar crest is a narrow ridge that separates the fenestra vestibuli from the horizontal semicircular canal.

The prootic is anteriorly flattened, with a small anteroventral suture to the basal plate of the parasphenoid. The prootic fails to reach the dermatocranium. Anteriorly the prootic is ventrally-deflected, contacting the pleurosphenoids at their lateral-most margin and providing the lateral wall for the trigeminal nerve foramen (Fig 6C). The prootic is medially flat, with its contribution to the dorsal shelf slightly separated from the suraoccipital. The fenestra vestibuli begins midway through the prootic and occupies most of the ventral margin of the prootic. Laterally the prootic is completely obscured from view by the squamosal and tabular.

The opisthotic meets the prootic in a straight articulation (Fig 6A and 6B). It mirrors the prootic, and has a ventral deflection at its posterior, and is slightly rounded where it encases the fenestra vestibuli. The opisthotic is similar in height to the prootic, but unlike its counterpart it does reach the dermatocranium, and is overlapped by the postparietal. The dorsal shelf of the opisthotic completely articulates with the supraoccipital. Posteriorly it sutures to the basioccipital ventrally and the exoccipital dorsally. At the junction between these elements is the jugular foramen. The opisthotics are visible in both lateral and posterior views.

The stapes nearly entirely fills the fenestra vestibuli, with little to no space at its margins (Fig 6A). The footplate is ovoid and slightly concave medially. The columella is short, robust, and anterolaterally oriented. It is directed towards the quadrate however the two are separated by the quadrate ramus of the pterygoid. The base of the columella is pierced by the stapedial foramen.

#### Lower Jaw

The lower jaw is composed of a dentary, prearticular, articular, angular, surangular, and two coronoids (Fig 5A and 5D). No sign of a splenial or postsplenial is present. The lower jaw is long, extending posteriorly to the occipitals, with the articulation just anterior to the otic capsules. It is narrow anteriorly, with a broad postdentary region that ends in a rounded edge slightly anterior to the occiput. The symphysis is composed solely of the dentary. The dentary is long, its width constant throughout. The coronoid process is upturned, extending slightly past the level of the postorbital. There are approximately 19 peg-like teeth on the dentary, the anterior 15 large and the last four small. The tooth row ends at the anterior margin of the dorsal coronoid prominence.

There are two coronoids in the lower jaw. The anterior coronoid is located anterior to the prearticular. It is a small element, its length spanning the level of teeth seven through nine of the dentary. The posterior coronoid is longer and broader, beginning at the level of the 14^th^ tooth and ending midway through the coronoid process. There is no lateral exposure of the coronoids, nor are there any denticles present. The prearticular is long, running from the posterior edge of the anterior coronoid to just past the midpoint of the coronoid process. The angular is of similar length, its anterior edge slightly posterior to that of the prearticular, sharing a posterior edge with the articular. The angular is somewhat broad and reaches the midline of the dentary. The surangular is almost twice as tall, dorsally bordering the angular and nearly reaching the coronoid process. It is convex, giving the posterior portion of the lower jaw a rounded shape. The coronoid process overlaps both angular and surangular, obscuring them from lateral view. The articular is a robust triangular bone with a process that fits into the quadrate in a saddle joint. There is a large, straight retroarticular process.

The Meckelian fossa is a triangular space, much narrower than seen in *Carrolla* [16]. Anteriorly it tapers to a point at the level of the third tooth, and remains narrow until the coronoid process of the dentary, after which it expands to fill the space of the posterior jaw. The Meckelian fossa is ventrally bordered by the dentary between teeth three through ten, and the angular provides the remainder of the ventral border. A medially oriented shelf on the dentary below the tooth row dorsally covers the Meckelian fossa from its anterior tip until the coronoid process, where it is dorsally bordered by the coronoid. Posterior to the coronoid process, the Meckelian fossa is laterally covered by the surangular and angular, and is medially covered by the prearticular. The Meckelian fossa is posteriorly bordered by the articular. A single foramen for the Meckelian fossa is present just posterior to the coronoid process of the dentary, between the angular and surangular.

### Description of *Aletrimyti gaskillae*

#### General

**Fig 7:**
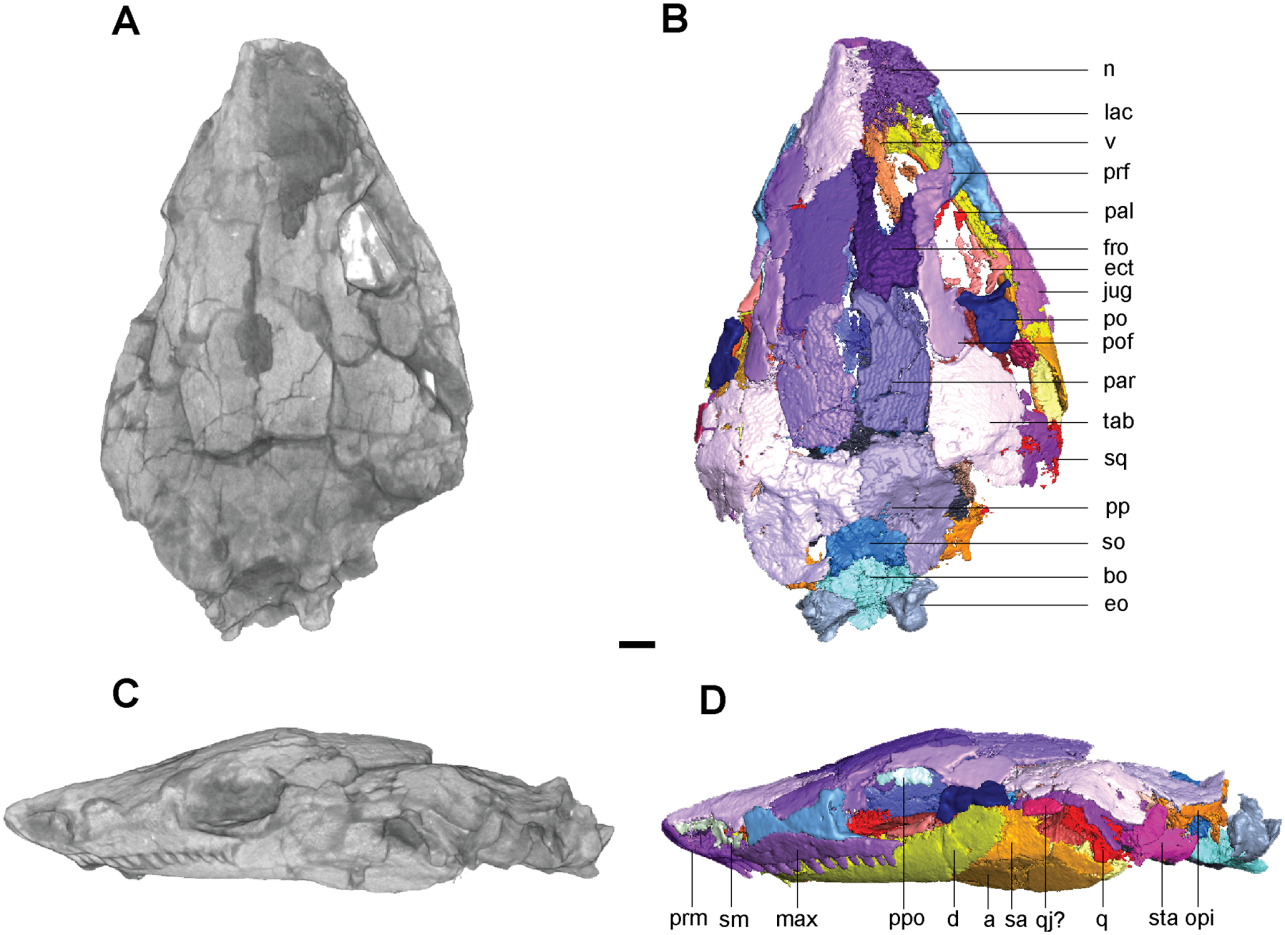
Volume renderings and volumizations of *Aletrimyti gaskillae*, *gen*. *et sp*. *nov*. (FM-UR 1040). Shown in dorsal view (**A**, **B**) and left lateral view (**C**, **D**). Volume renderings (**A**, **C**) depict the specimen after being submitted to HRXCT. Volumizations (**B**, **D**) show the bony elements after they have been isolated. Scale bar equals 1mm. Abbreviations: a, angular; **bo**, basioccipital; **d**, dentary; **ect**, ectopterygoid; **eo**, exoccipital; **fro**, frontal; **jug**, jugal; **lac**, lacrimal; **max**, maxilla; **n**, nasal; **opi**, opisthotic; **pal**, palatine; **par**, parietal; **po**, postorbital; **pof**, postfrontal; **pp**, postparietal; **ppo**, palpebral ossification; **prf**, prefrontal; **prm**, premaxilla; **pte**, pterygoid; **q**, quadrate; **qj**, quadratojugal; **sa**, surangular; **sm**, septomaxilla; **so**, supraoccipital; **sq**, squamosal; **sta**, stapes; **tab**, tabular; **v**, vomer.

The skull of FM-UR 1040 is well-preserved with few deformations (Fig 7A and 7C). The specimen is approximately 19mm in length, 11mm wide at the parietals, and 5mm tall at the orbit. The right nasal and right palate are only partially preserved; there has been a slight crushing in the postparietal region, and the lower jaw has been shifted right-laterally. The orbit is circular on the left side, and, due to deformation, slightly elliptical on the right. The nasal has collapsed to fill the external naris on the right side, but is preserved on the left. The jaws are preserved in tight articulation, with the coronoid process of the left dentary pushed into the postorbital. The jugal is missing on the left side where the jaw has been laterally shifted left. No sculpture or ornamentation of the bone is present.

Seen dorsally the skull (Fig 7A and 7B) is diamond-shaped, with a pointed snout, broadest at the level of the parietals and quadrates, quickly narrowing in the otic capsules and occipitals. The nasals, frontals, and parietals are all roughly equal in length and width. The right orbit is visible in dorsal view, whereas the left one is obscured due to the rightward shift of the lower jaw. In lateral view the skull is long and short (Fig 7C and 7D), tapering at both anterior and posterior ends; its highest point is the posterior margin of the orbit. The snout morphology is consistent with that described as shovel-headed in amphisbaenids [25], and is slightly upturned. There is a slight vertical gap between the parietals and postparietals where the posterior portion of the skull has been crushed.

The skull of UCMP 202927 has been heavily crushed. The dorsal elements of the skull roof are relatively well preserved, as are the palatal elements, whereas the remainder of the skull is distorted. As such, only a few elements are called out in the description of *Aletrimyti gaskillae*, gen. et sp. nov., supplementing the holotype.

#### Skull Roof

The premaxilla is a tall recumbent element, nearly as wide as the nasals when seen dorsally, and half their length (Fig 7B and 7D). It has a dorsal articulation with the nasal that gives the snout its sharp, upturned shape. The premaxilla lacks a dorsal exposure and is completely obscured by the nasal. The external naris on the left side is almond-shaped, the long axis orientated horizontally, with the premaxilla forming the anteroventral half of the border. The premaxilla bulges over the tooth row, medially creating a sinus for the nasal capsule with the nasal bone, and forming the anterior border of the choana. There are five large, peg-like teeth on the premaxilla.

**Fig 8:**
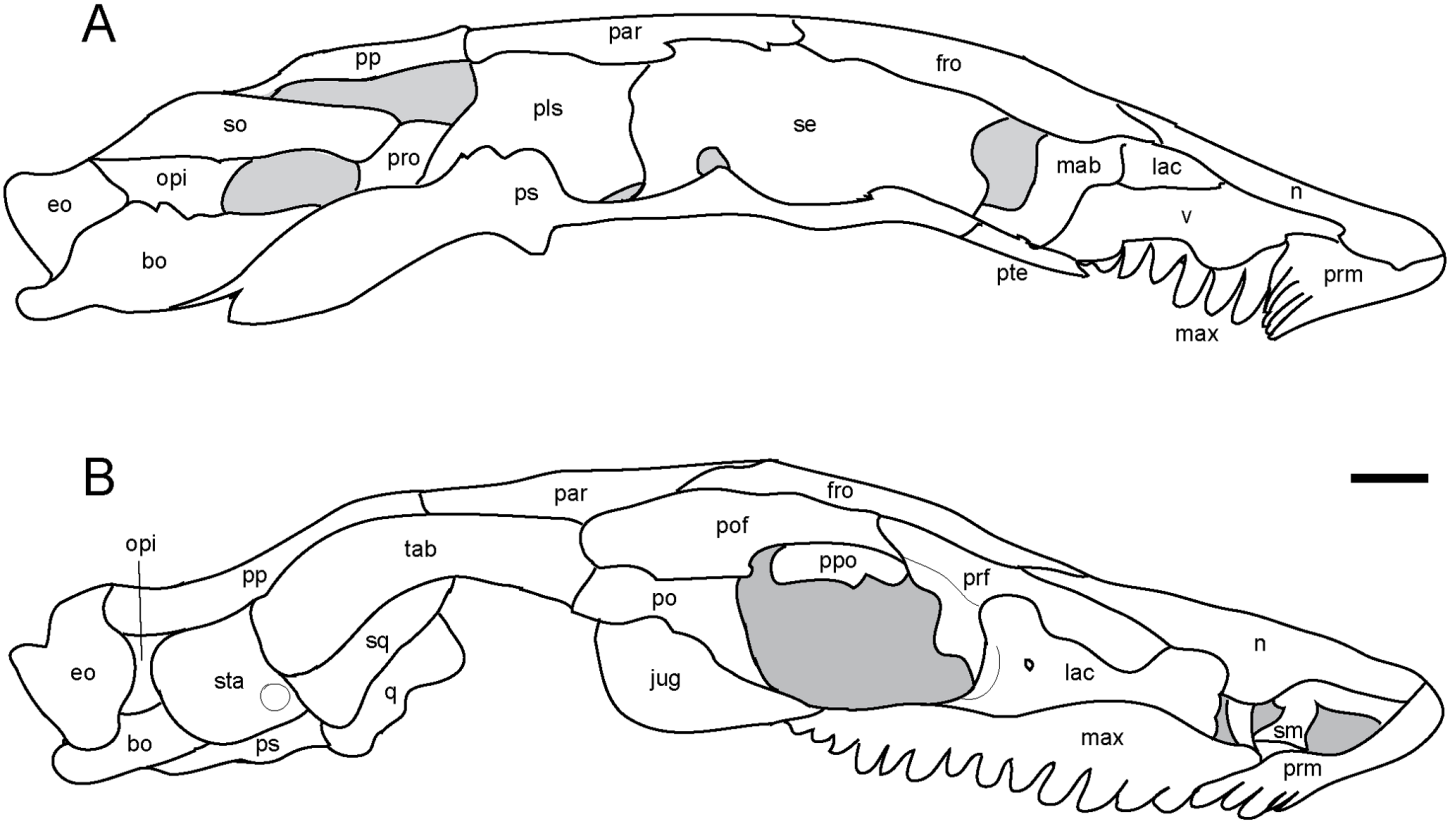
Interpretive drawings of *Aletrimyti gaskillae*, *gen*. *et sp*. *nov*. (FM-UR 1040). **A**, medial view; **B**, right lateral view. Lower jaw has been removed to better reveal palate and cheek elements. Scale bar equals 1mm. Abbreviations: **bo**, basioccipital; **eo**, exoccipital; **fro**, frontal; **jug**, jugal; **lac**, lacrimal; **mab**, ‘median anterior braincase bone’; **max**, maxilla; **n**, nasal; **opi**, opisthotic; **par**, parietal; **pls**, pleurosphenoid; **po**, postorbital; **pof**, postfrontal; **pp**, postparietal; **ppo**, palpebral ossification; **prf**, prefrontal; **prm**, premaxilla; **pro**, prootic; **ps**, parasphenoid; **pte**, pterygoid; **q**, quadrate; **se**, sphenethmoid; **sm**, septomaxilla; **so**, supraoccipital; **sq**, squamosal; **sta**, stapes; **tab**, tabular; **v**, vomer.

A septomaxilla is located within the left naris. It is medially convex and thin, spanning from the anterodorsal corner of the naris to the posteroventral corner. A small fragment of bone at the posteroventral corner of the naris, interpreted as a lateral exposure of the septomaxilla, articulates with the posterior margin of the premaxilla and a descending process from the nasal to close the naris, precluding the lacrimal from bordering the naris (Fig 7D and Fig 8B).

The nasal is long and pentagonal in dorsal view. The anterior margin is straight where it covers the premaxilla, and tapers to a point at the posterolateral margin where it lies between the frontal and prefrontal (Fig 7B). Posteriorly the nasal slightly overlaps the frontal, and is overlapped posterolaterally by the prefrontal and at a small lateral section by the lacrimal. Each of these overlaps is accommodated by a facet on the nasal. A small flange descends from the nasal containing the facet for the lacrimal, and meets the septomaxilla midway through the naris, excluding the prefrontal from bordering the naris. The nasal forms the posterodorsal half of the border of the external naris.

The frontal is a long, parallelogram-shaped bone (Fig 7B). The pointed anterior is slightly overlapped by the nasal. Posteriorly it overlaps the parietal, forming a scarf joint. Anterolaterally the prefrontal rests atop a notch in the frontal and the postfrontal does the same posterolaterally. A flange on the underside of the frontal runs along the lateral margin, beginning midway through the frontal and extending posteriorly (Fig 8A). This flange descends to meet the sphenethmoid and rest within a lateral embayment of the latter. The flange nearly spans the length of the orbit before shifting laterally and out of articulation with the sphenethmoid. Anteriorly this flange is continuous with the contour of the descending flange of the prefrontal; however, the two do not meet and instead there is a notch between them, interpreted as for the deep ophthalmic branch of the trigeminal nerve.

The parietal is a long rectangular bone. A facet on the anterior margin is overlapped by the frontal, making the parietal tapered in dorsal view (Fig 7B). The parietal contacts the postfrontal laterally with a small facet for the postfrontal to overlap. The lateral and posterior margins of the parietal are flat, and they meet the tabular and postparietal (respectively) in butt joints. In UCMP 202927 a small pineal foramen is present on the midline suture, at the anterior third of the parietals (Fig 2C and 2D). In ventral view, a longitudinal flange on the parietal is continuous with that of the frontal, with only a small gap separating the two. This flange continues until approximately midway through the parietal, where it turns medially and the two sides connect just posterior to the pineal foramen. Seen on the underside of the parietals, the pineal foramen is at the anterior margin of a deep, circular fossa for the pineal organ (Fig 2C and 2D).

On the ventral margin of the frontals and parietals, medially between the flanges, is a depression interpreted to reflect the dorsal extent of the brain, spanning the length of the flanges (Fig 2C and 2D). This depression is somewhat elliptical; the posterior end is rounded and the anterior end is a double arch. The walls lateral to this depression formed by the flanges of the frontals and parietals are anteroposteriorly straight.

The postparietal is a large, rounded element with a straight anterior margin. Posteromedially at the midline suture there is a space between the postparietals that dorsally exposes the supraoccipital (Fig 7B). The postparietal is slightly ventrally sloped where it covers the occiput, with a well-defined nuchal ridge between the flat skull table and the sloped occiput. Both postparietals have been ventrally shifted; a gap is visible between the posterior edge of the parietals and the anterior edge of the postparietals. This shift has caused them to completely cover the otic capsules dorsally, although there was most likely a small sinus ventral to the flat dorsal portion of the postparietals. A small facet on the anterior edge of the postparietal would have accommodated an overlapping parietal. Laterally it is overlapped by the tabular.

The tabular is a large, round element, slightly larger than the orbit. Its anterior surface is concave where it houses the rounded posterior of the postfrontal, which overlaps a small shelf of the tabular. Medially it abuts the parietal and posteromedially it overlaps the postparietal. The cheek has been dorsally shifted on the left, and the tabular nearly completely overlaps the squamosal, leaving only a small ventral exposure. The likely natural condition is seen on the right (Fig 7B), where only a small margin is overlapped. The posterior edge of the tabular is exposed, and is without articulation.

The squamosal is a small triangular bone, approximately half the size of the tabular. It is slightly overlapped dorsally by the tabular on the right and greatly on the left where the cheek has been shifted dorsally (Fig 7B and 7D). Medially the squamosal overlaps the quadrate. The anterior margin of the squamosal borders the ventral cheek emargination, and it fails to reach the postorbital. No otic notch is present.

#### Circumorbitals

A raised ridge of bone is present along the margins of the orbit. The large, circular orbits each occupy approximately 17% of the area of the skull (Fig 7D).

The lacrimal forms the anterior margin of the orbit. It is a long, hourglass-shaped bone that nearly reaches the naris, with a suborbital process nearly reaches the jugal (Fig 7D). Anteriorly, a smooth facet on the nasal accepts the smaller overlapping anterior process of the lacrimal. A dorsally-oriented medial flange from the lacrimal forms the antorbital wall. This flange extends medially to the palatine, and overlaps the descending medial flange of the prefrontal. Two foramina are present on the antorbital wall. An anteriorly-oriented foramen is interpreted to be an orbital opening of the nasolacrimal duct, while a second, laterally-oriented foramen is interpreted as the lacrimal canal.

The prefrontal forms the anterodorsal margin of the orbit. It is a teardrop-shaped element that extends from the midpoint of the lacrimal to the orbital dorsal midpoint (Fig 7D). The broad anterior margin of the prefrontal is nestled between the nasal, which it overlaps, and the lacrimal, which it underlaps. A narrow process meets the postfrontal in a scarf joint midway through the orbit to form the anterior half the dorsal margin of the orbit. A descending medially-oriented flange participates in the antorbital wall, where it is overlapped by the flange of the lacrimal. Medially the prefrontal is smooth and concave, and forms the dorsal half with the lacrimal of a sinus for the vomeronasal organ and nasal capsule.

The postfrontal is also teardrop-shaped, and forms the posterodorsal margin of the orbit (Fig 7D). A narrow anterior process forms a scarf joint with the prefrontal above the midpoint of the orbit. The postfrontal’s rounded posterior end is bordered by the parietal, tabular, and postorbital, the latter of which sits atop a notch in the postfrontal. Small grooves in the parietal and tabular accept the postfrontal. The anterior process is slightly overlapped by the frontal, for which there is a small notch.

The postorbital forms the posterior margin of the orbit. It is relatively small, approximately half the size of the postfrontal (Fig 7D). It overlaps the postfrontal dorsally at a small facet. The raised ridge of bone surrounding the orbit is most pronounced on the postorbital. The postorbital posteriorly borders the ventral cheek emargination.

The jugal is a triangular bone, longer than the postorbital but not quite as long as the postfrontal (Fig 7D). It forms the posteroventral margin of the orbit. There is a slight anterior overlapping of the maxilla, which has a smooth facet that accepts the jugal. Anteriorly it nearly reaches the suborbital process of the lacrimal, leaving a small exposure of the maxilla that forms the remainder of the ventral edge of the orbit. A small notch midway along the dorsal edge of the jugal may have accepted the raised anterior ridge of the postorbital. Posterior to this notch the jugal is flat, with no evidence of either under- or overlapping the postorbital. The ventral cheek emargination separates the jugal from the squamosal.

Posterior to the left postorbital is a small fragment of bone (Fig 7D). It is straight and slender, approximately half the length of the postorbital, and a quarter of its width. This fragment is preserved lateral to the posterior lower jaw elements, and ventral to the squamosal. A larger fragment is located posterior to the right postorbital. It is approximately half the length and width of the postorbital, and is located ventral to the tabular, between the postorbital and squamosal. Unlike the fragment on the left, the one on the right is medial to the posterior lower jaw elements. These two fragments represent the only possible evidence of a quadratojugal.

The ventral cheek emargination is bordered dorsally by the tabular, posteriorly by the squamosal, and anteriorly by the postorbital and jugal (Figs 7C, 7D and 8B). The transverse process and quadrate ramus of the pterygoid form the medial border of the cheek emargination. The cheek emargination is similar to that seen in *Pelodosotis* [19] and other ostodolepids, although not quite as large, and may have provided a space for the adductor musculature to bulge when contracted.

The maxilla is relatively short: its anterior is slightly ahead of that of the lacrimal, and it ends just past the midpoint of the orbit, tapering to where it underlaps the jugal (Fig 7B). Approximately 13–14 teeth are present. The non-pedicellate teeth are long, peg-like, and recurved, the first three large and the subsequent teeth gradually diminishing in size. A shelf along the medial surface of the maxilla articulates with the palatine and forms the lateral edge of the choana. The maxilla has a small overlap of the premaxilla anteriorly, at the posterior edge of the external nares, which it does not border. A small exposure of the maxilla contributes to the ventral margin of the orbit.

A palpebral ossification is located within the dorsal margins of the left orbit (Fig 7D). It is a single, unbroken element that spans nearly the length of the dorsal margin. Only a sliver of the palpebral is visible in dorsal view (Fig 7B), and the rest is obscured by the pre- and postfrontals.

#### Palate

The vomer is long, anteriorly in contact with the premaxilla and overlapping the anterior margin of the pterygoid (Fig 3B). The vomer is not fully preserved, and would have been ovoid in shape when seen in ventral view, separated from the maxilla by the choana. The vomer is dorsally concave; its medial wall is upright while the lateral wall is splayed at an oblique angle. The choana is relatively large and elliptical, located posterior to the external naris. It is bordered medially by the vomer and spans nearly half the vomer’s length. The medial walls of the vomers meet to form a septum along the midline of the braincase, articulating with the ventral side of the nasals. It is important to note, however, that this articulation between vomers only occurs for the dorsal half of the medial walls, and the vomers do not contact more ventrally. No teeth are preserved on the vomers.

The palatine is similar in area to the vomer (Fig 3B). Anteriorly a U-shaped notch in the palatine between the medial vomer and lateral maxilla forms the posterior edge of the choana. The palatine contacts the maxilla laterally via a medially oriented shelf from the maxilla. Ventrally three conical teeth are preserved in an anteroposterior row, parallel to those on the maxilla.

The ectopterygoid is nestled posteriorly where the basipterygoid and palatine processes of the pterygoid meet (Fig 3B). Due to poor ossification it fails to reach the palatine anteriorly. Laterally its edge is straight and runs parallel to the maxilla; presumably the two would have been tightly sutured together. A row of four small teeth are preserved on the ectopterygoid, continuous with those on the palatine.

The pterygoid is the largest element of the palate, spanning from midway through the maxilla to midway through the basal plate of the parasphenoid, approximately half the length of the skull (Fig 3B). The palatine process is long and narrow, and forms the lateral margin of the narrow interpterygoid vacuity. A U-shaped notch between the transverse process and the palatine process accepts the ectopterygoid. A small gap separates the pterygoids at their anterior-most point where they articulate with the vomers. The pterygoid tapers at the basipterygoid process of the parasphenoid, widening the posterior half of the relatively narrow interpterygoid vacuity. The posterior half of the pterygoid is ventrally concave where the quadrate ramus joins the palatine process. The quadrate ramus is tall and flat, and extends posteriorly to lie flat against the medial side of the quadrate. Anterior to the quadrate and medial to the cheek emargination, the quadrate ramus is somewhat laterally concave, medially bounding the large subtemporal fenestra. Dorsal to the basipterygoid process the epipterygoid is tightly sutured to the pterygoid. No dentition is observed on the pterygoid.

Dorsal to the pterygoid, the epipterygoid is a square sheet of bone, similar in size to the quadrate ramus of the pterygoid. It rests atop the basipterygoid process of the parasphenoid, and has a tight ventral suture with the pterygoid, and a small posteroventral one with the basal plate of the parasphenoid (Fig 3B). It slightly overlaps the quadrate ramus of the pterygoid laterally, obscuring the anterior-most margin of the quadrate ramus from lateral view. Posterolaterally a process fits into a notch at the anteromedial margin of the quadrate, and would have joined the quadrate and epipterygoid.

The quadrate is a robust, pyramidal bone with a concave anterior face (Fig 5B). Its point is vertically oriented, and the dorsal margin of the quadrate meets the tabular. In dorsal view the quadrate is triangular, with points oriented anterolaterally, anteromedially, and posteriorly. A process extending from the epipterygoid faces a facet on the anteromedial margin of the quadrate, separated by a small gap, and the two would have likely met. Medially the quadrate is tightly sutured to the quadrate ramus of the pterygoid, which medially covers much of the quadrate, leaving only a small posterior exposure. There is a small gap between the stapes and the quadrate posteromedially and the two may have articulated. Laterally the quadrate is completely overlapped by both the tabular and squamosal. Ventrally the trochlear surface of the quadrate is preserved, which forms a saddle joint with the articular (Fig 5E).

#### Braincase and Occiput

**Fig 9:**
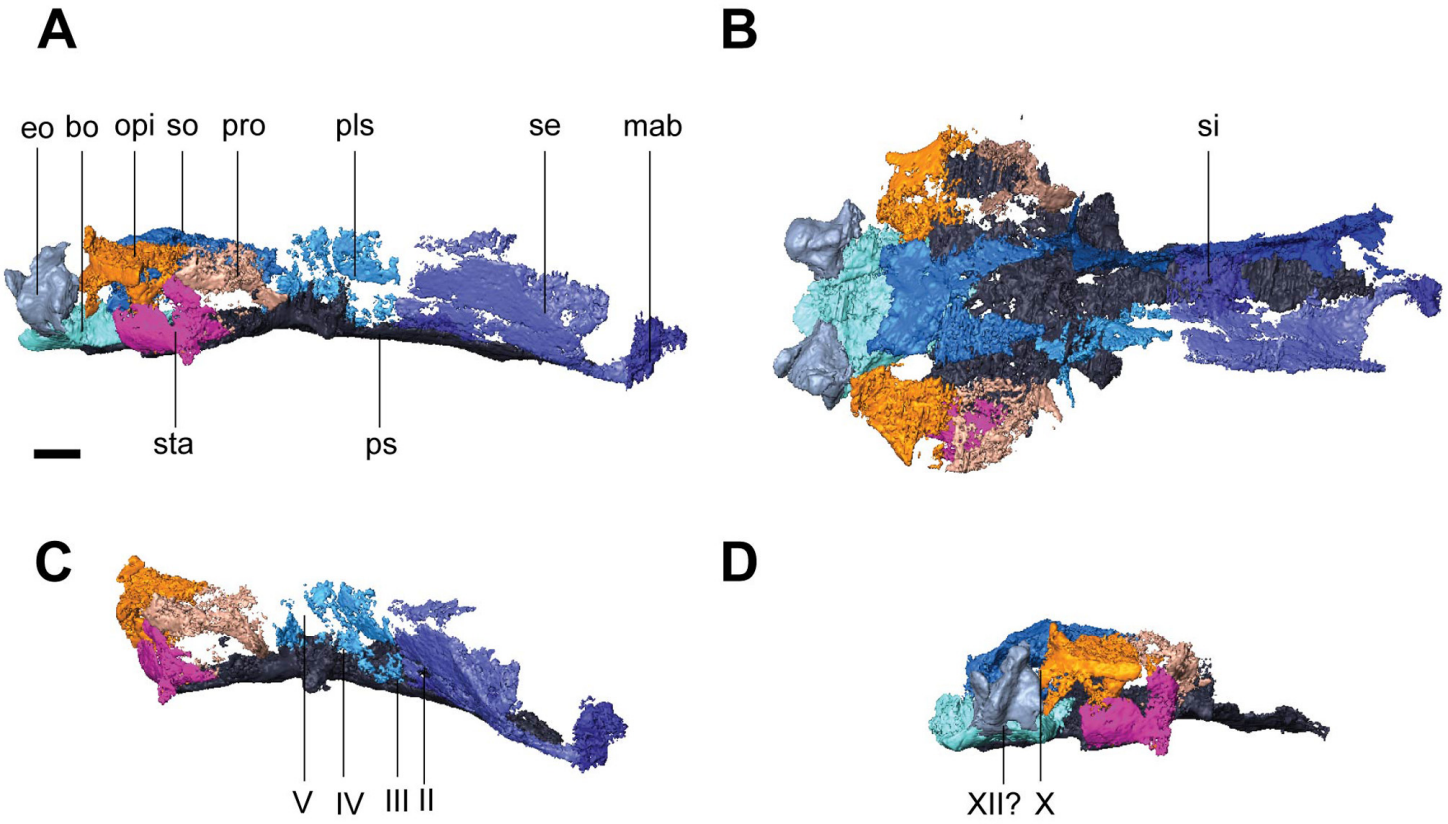
Isolated braincase elements of *Aletrimyti gaskillae*, *gen*. *et sp*. *nov*. (FM-UR 1040). Shown in right lateral (**A**), dorsal (**B**), right anterolateral oblique (**C**), and right posterolateral oblique (**D**) views. Surrounding elements have been removed to expose braincase. Scale bars equal 1mm. Abbreviations: **bo**, basioccipital; **eo**, exoccipital; **mab**, ‘median anterior braincase bone’; **opi**, opisthotic; **pls**, pleurosphenoid; **pro**, prootic; **ps**, parasphenoid; **se**, sphenethmoid; **si**, ossification of the subiculum infundibulum; **so**, supraoccipital; **sta**, stapes; **II**, optic nerve foramen; **III**, oculomotor nerve foramen; **IV**, trochlear nerve foramen; **V**, fenestra prootica; **X**, foramen for the vagus nerve and jugular vein; **XII**, hypoglossal nerve foramen.

The braincase is long and slender, beginning at the anterior margin of the orbit and extending to the posterior edge of the parietals, and dorsally meets the dermatocranium (Figs 8A and 9A). It is slightly anteriorly sloped when seen in lateral view.

The parasphenoid has a broad posterior basal plate with a relatively broad cultriform process (Fig 3B). The cultriform process extends from the anterior margin of the orbit to just past the midpoint of the parietals, and has a rounded anterior tip. The parasphenoid is horizontally level with the palatal elements. A small patch of denticles is present at the base of the cultriform process. The cultriform process is slightly concave dorsally where it receives the sphenethmoids. At the base of the cultriform process are the basipterygoid processes, medial to which there is a slight dorsal recess for the hypophysial fossa (Fig 9B). Ventral to each basipterygoid process is an arched groove that runs from anterior to posterior edges of the process, interpreted as the vidian sulcus. The vidian sulcus is continuous along the length of the cultriform process, where grooves are present along each lateral margin for the palatal branches of the internal carotid arteries. An anterolaterally oriented groove on the anterodorsal side of each basipterygoid process leads to a foramen for the trochlear nerve in the pleurosphenoids (Fig 9C). The basal plate of the parasphenoid broadens posteriorly from the basipterygoid processes, widening to underplate the otic capsules before narrowing as it underplates the basioccipital, where there is a recess for the basicranial fenestra. The basal plate is somewhat pentagonal with a posteriorly-directed point.

The sphenethmoids are long and rectangular when seen in lateral view (Figs 8A and 9A). They extend anteriorly slightly past the cultriform process, and their posterior edge is anterior to the basicranial articulation. They provide the lateral walls to the anterior braincase. The sphenethmoids have a small ventromedial keel-like articulation with a distinct suture at the anterior margin of the cultriform process of the parasphenoid. They reach the dermatocranium dorsoventrally, where the ventral flange of the frontals fit into the sphenethmoids. This articulation is approximately half the length of the orbit. The descending flange of the parietal does not articulate with the sphenethmoid, but is instead separated from the sphenethmoid by a narrow gap for the remainder of the sphenethmoid. The optic foramen is preserved at the posterior portion of the sphenethmoids, entirely housed by the sphenethmoids, slightly anterior to the pleurosphenoids (Fig 9C).

Ventromedial to the sphenethmoids is an ossification of the subiculum infundibuli (Fig 9B). This ossification shares a posterior border with the sphenethmoids and extends to just anterior of the optic foramen. Anterior to the ossification is a recess in the parasphenoid interpreted as for an extension of the ventral brain.

Posterior to the sphenethmoids are the pleurosphenoids. They are approximately half the length of the sphenethmoids, and dorsally span from the parasphenoid to the dermatocranium. Located at the anterior margin of the pleurosphenoids is the oculomotor foramen (Fig 9C). Posterior to this is an anterolaterally directed foramen dorsally bordered by the pleurosphenoids and ventrally bordered by the basal plate of the parasphenoid, at the level of the basipterygoid processes, interpreted as the foramen for the trochlear nerve (Fig 9C). The pleurosphenoids do not reach the occiput; instead, a space between the dorsum sellae and the basioccipital is interpreted as the basicranial fenestra. A laterally oriented process extends from the posterior margin of the pleurosphenoid towards the prootic, articulating with a similar process from the prootic. Dorsal to this process is a large foramen, interpreted as the fenestra prootica, allowing for the passage of the trigeminal nerve (Fig 9C).

Ventromedially connecting the sphenethmoids anteriorly in UCMP 202927 is a separate ossification in the ethmoid region, similar to the ‘median anterior braincase bone’ in *Carrolla* (Figs 8A and 9A–9C). It stretches from the anterior tip of the cultriform process to the posterior margin of the nasal where it meets the dermatocranium, and has a large base with a Y-shaped dorsal projection. Laterally it is slightly concave, and with the vomers may have formed the internarial septum.

The occipital region is composed of a basioccipital, paired exoccipitals, and a supraoccipital (Fig 9B and 9D). Together these elements border the foramen magnum. The basioccipital is broad, slightly narrower than the lateral margins of the basipterygoid processes, and long, extending from the midpoint of the basal plate of the parasphenoid to posteriorly past the occipital condyles of the exoccipitals. The paired exoccipitals are tall, round elements that form the occipital condyles. The occipital condyles are well developed, and flank the posteriorly convex occipital cotyle that forms a deep recess for the atlas, similar in shape to the complex seen in *Carrolla*. The exoccipitals overlap the basioccipital posteriorly. A large foramen, interpreted as the jugular foramen, is visible on the lateral margin, anterior to the occipital condyles between the exoccipitals and the opisthotics (Fig 9D). A smaller foramen is also visible ventral to the occipital condyles, bordered by the basioccipital, possibly the hypoglossal foramen (Fig 9D). The supraoccipital is partially concealed by the postparietals. It has been shifted anteriorly out of articulation with the exoccipitals, but would have contacted them. It is arched, enclosing the foramen magnum. A pair of long, lateral processes extends anteriorly from the supraoccipital that, due to the anterior shift of the supraoccipital, nearly contact the pleurosphenoids. There is a small sinus between the supraoccipital and the postparietals, similar to that in *Pelodosotis*.

#### Otic Capsules

The otic capsules are composed of a distinct anterior prootic and a posterior opisthotic, both of which are equal in size. They are robust, beginning posterior to the basipterygoid processes of the parasphenoid and extend posteriorly past the posterior margin of the basal plate of the parasphenoid to the exoccipitals. The otic capsules are relatively wide, each nearly spanning from the lateral margin of the basal plate of the parasphenoid, nearly reaching the midline. The otic capsule is arched where it encloses the fenestra vestibuli and the stapes. A medially oriented, flat shelf formed by the prootic and opisthotic covers the dorsal surface of the otic capsule. It appears as though this shelf would have articulated with the lateral processes of the supraoccipital, where slight medial notches in the opisthotics match the lateral edge of the lateral processes. The otic capsules reach the dermatocranium dorsally, and the dorsal shelf sutures to the postparietals, with a small sinus dorsal to the prootic. Ventrally the otic capsules have only a small point of contact with the parasphenoid anteriorly; posteriorly the otic capsules contact the basioccipital and exoccipitals. In dorsal view the otic capsules are laterally convex and medially straight. They extend slightly past the lateral margin of the basal plate of the parasphenoid. Ventral to the dorsal shelf the medial surface is concave and smooth. Here the margins for the horizontal semicircular canal are preserved, encapsulated by both elements of the otic capsule. Ventral to the horizontal semicircular canal is the lagenar crest, a narrow ridge separating the semicircular canal from the fenestra vestibuli.

The prootic is anteriorly rounded, with a small anteroventral suture to the basal plate of the parasphenoid (Fig 9C). Between the dorsal margin of the prootic and the postparietal is the dorsal occipital sinus (Fig 8A). Anteriorly an anterolaterally oriented process extends from the prootic. This process is matched by the posterior process of the pleurosphenoid, and the two articulate at their respective extremities. The medial margin of the prootic is straight, and does not appear to have articulated with the supraoccipital. Midway through the fenestra vestibuli, the posterior margin of the prootic is straight where it articulates with the opisthotic. Laterally the prootic is completely obscured from view by the tabular (Fig 8B).

The opisthotic is anteriorly straight, and in tight articulation with the prootic. The opisthotic is ventrally rounded, similar to the prootic (Fig 9A and 9D). Posteriorly the opisthotic is flat ridge that contacts the basioccipital ventrally and the exoccipital dorsally. This ridge provides the anterior margin for the jugular foramen at the posterodorsal margin of the opisthotic. Medially, a small notch in the dorsal shelf would have accepted the lateral process of the supraoccipital, which would have been in articulation for the length of the opisthotic.

The stapes occupies nearly the entirety of the fenestra vestibuli, leaving a space between the anterior- and posterior-most margins. The footplate of the stapes is ovoid and slightly concave medially. The columella is short, robust, and anterolaterally oriented. It is directed towards the quadrate and the two likely came into contact. The base of the columella is pierced by the stapedial foramen. There is no evidence of an accessory ossicle; rather, the accessory ossicle described by Carroll and Gaskill [19] appears to be a fragment of the tabular, ventrally deflected and preserved in contact with the left stapes (Fig 9D).

#### Lower Jaw

The lower jaw is composed of a dentary, prearticular, articular, angular, surangular, splenial, postsplenial, and coronoid (Fig 5B and 5E). It is a long element that extends from the anterior margin of the skull to the otic capsules, just anterior to the occiput. It is narrow at the symphysis, broadens at the coronoid process of the dentary (where the dentary meets the surangular), and narrows again towards the articular. The dorsal edge of the surangular is slightly rounded, giving the lower jaw a hyperbolic shape in lateral view. The symphysis is composed of the dentary and splenial bones. The dentary is a long, thin element that is narrow at the symphysis and has a broad, upturned coronoid process that extends posteriorly past the orbit and ends at the level of the posterior margin of the postorbital. There are approximately 18–19 peg-like teeth on the dentary, which are larger anteriorly and become smaller posteriorly. The tooth row ends at level of the anterior margin of the surangular.

The coronoid is long, beginning at the eighth tooth of the dentary and running posteriorly the rest of the length of the dentary. There is a small lateral exposure of the coronoid, anteroventral to the cheek emargination. A few denticles are present on the coronoid. The splenial contributes to the symphysis of UCMP 202927. It is long, spanning the length of the first ten teeth of the dentary, with a slightly tapered anterior. A long post-splenial runs medial to the prearticular, ending at posteriorly at a level equal to the coronoid process of the dentary. The post-splenial is separated from the splenial by a small gap, with no contact between the two. Between the angular and the coronoid is the prearticular. It begins at the level of the eighth tooth of the dentary and extends posteriorly to the anterior edges of the quadrate and the articular bones. The angular begins at the level of the eighth tooth of the dentary and spans the rest of the length of the lower jaw, creating the ventral surface for most of the jaw. It is tall, reaching dorsally to the approximate midpoint of the dentary. Dorsal to it is the surangular, which has a dorsal edge that reaches the coronoid process of the dentary, and is slightly concave. Both angular and surangular cover the articular laterally, and are both overlapped by the coronoid process. The articular is small, with a deep groove that accepts the quadrate. The articular is walled laterally by the surangular and angular, medially by the prearticular, and is floored by the angular. The articular is concave where it articulates with the quadrate. A large retroarticular process is preserved on UCMP 202927, extending posteriorly to midway through the otic capsule. It is long and straight.

The Meckelian fossa is a triangular space, much narrower than in *Carrolla*. Anteriorly it is tapered to a point, and maintains its narrow width until the coronoid process of the dentary where it broadens to fill the space of the posterior jaw. The fossa nearly reaches the symphysis, beginning at the level of the second tooth. It is ventrally bordered by the dentary between teeth two through eight, after which it is floored by the angular. A medially oriented shelf extends below the tooth row of the dentary and provides the dorsal border for the Meckelian fossa. At the end of the tooth row it expands to fill the posterior jaw, where it is laterally bordered by the angular and surangular. The splenial provides the medial wall for the Meckelian fossa between teeth two through eight, while the prearticular provides the medial wall for the Meckelian fossa from tooth eight to the articular. No foramen for the Meckelian fossa is present.

### Description of *Dvellecanus carrolli*

#### General

**Fig 10:**
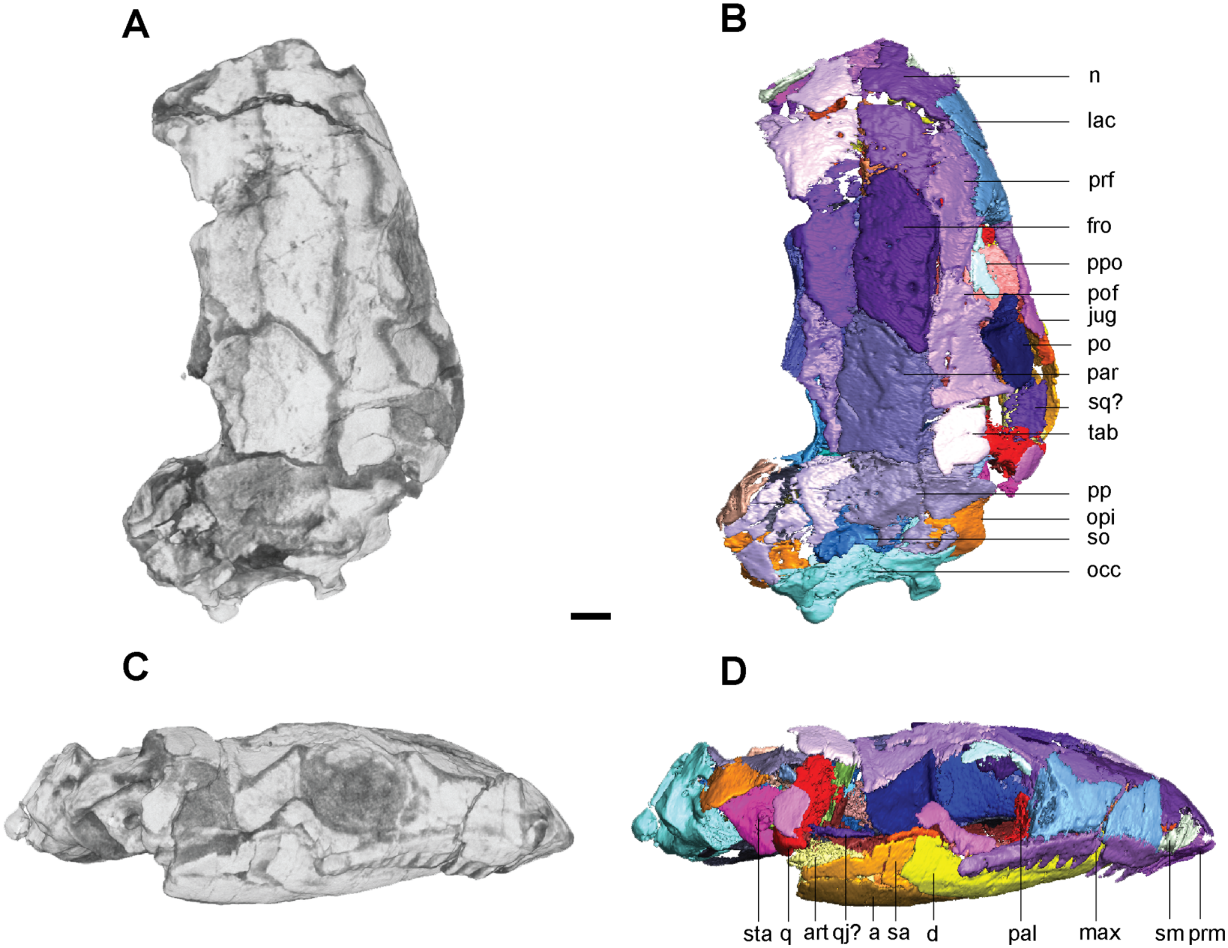
Volume renderings and volumizations of *Dvellecanus carrolli*, gen. et sp. nov. (UCMP 202940). Shown in dorsal view (**A**, **B**) and right lateral view (**C**, **D**). Volume renderings (**A**, **C**) depict the specimen after being submitted to HRXCT. Volumizations (**B**, **D**) show the bony elements after they have been isolated. Scale bar equals 1mm. Abbreviations: **a**, angular; **d**, dentary; **fro**, frontal; **jug**, jugal; **lac**, lacrimal; **max**, maxilla; **n**, nasal; **occ**, occipital complex; **opi**, opisthotic; **pal**, palatine; **par**, parietal; **po**, postorbital; **pof**, postfrontal; **pp**, postparietal; **ppo**, palpebral ossification; **prf**, prefrontal; **prm**, premaxilla; **pte**, pterygoid; **q**, quadrate; **qj**, quadratojugal; **sa**, surangular; **sm**, septomaxilla; **so**, supraoccipital; **sq**, squamosal; **sta**, stapes; **tab**, tabular.

The left side of the skull of the holotype has been removed previously via preparation to expose the braincase (Fig 10A and 10C) [19]. The skull is approximately 14mm in length, 6mm wide between midline and lateral edge, and 4mm tall at the orbit. The squamosal is missing completely, laterally exposing the quadrate. The dermal bone is smooth, with no sculpturing or ornamentation. In dorsal view the skull is roughly egg-shaped, with a narrow, rounded snout, broadening at the posterior margin of the parietals and rounding at the occiput (Fig 10A and 10B). The nasals, frontals, and parietals are all of equal width; however, the nasals are shorter than the frontals and parietals, which are of equal length. When seen in profile the skull is triangular (Fig 10C and 10D). The snout is pointed and downturned, consistent with morphology termed ‘spade-headed’ in amphisbaenids [25]. The external nares are elliptical, with the long axis orientated horizontally. The right orbit is large and circular. The jaws are preserved in tight articulation, presumably in their proper orientation.

#### Skull Roof

The premaxilla is short and recumbent, meeting the downward sloped nasal in a point (Fig 10C and 10D). The nature of the suture leaves no dorsal exposure of the premaxilla. The premaxilla is almost as wide as the nasal. The elliptical external naris is anteroventrally bordered by the premaxilla. Where the premaxilla extends anteriorly over the tooth row it creates a medial sinus for the nasal capsule, along with the nasal bone. The tooth row forms the anterior margin of the choana. The premaxilla contacts the lacrimal posteriorly at a facet along the lateral edge filled by the septomaxilla. There are five large, peg-like, slightly recurved teeth preserved on the premaxilla.

A crescent-shaped septomaxilla is preserved within each naris, lying within a facet on the premaxilla. It spans the interior length of the naris, from the anterior joining of the premaxilla and nasal to the posterior lacrimal (Fig 10D).

The nasal is a long, parallelogram-shaped element. The nasal forms the dorsal half of the border for the external naris, with a descending process at the posterior of the external naris that precludes the lacrimal from the dorsal margins of the naris (Fig 10B). Laterally the nasal is overlapped by the lacrimal anteriorly and the prefrontal posteriorly, a facet in the nasal accommodating each of these elements. Aside from a slight overlap at the posterolateral point, the nasal abuts the frontal.

The frontal is long and parallelogram-shaped. The lateral margin is long and straight, and bears a slight facet to receive the overlapping pre- and postfrontals (Fig 10B). Anteriorly the lateral portion of the frontal is overlapped by a process from the nasal, forming a partial scarf joint, while the anteromedial portion abuts the nasal in a straight edge. The entire posterior portion of the frontal overlaps the parietal in a scarf joint. A flange descends from the frontal ventrally, extending along the lateral margin of the frontal, from the antorbital wall to the posterior edge of the frontal. A small facet on the dorsal margin of the sphenethmoid accepts this flange for nearly the length of the orbit, at which point the flange redirects posterolaterally. The flange follows the contours of the descending flange of the prefrontal, but the two do not come into contact; the small gap between the two is interpreted as for the ophthalmic branch of the trigeminal nerve, as in *Rhynchonkos* and *Aletrimyti*.

The parietal is trapezoidal with an anterior margin that comes to a point at the midline (Fig 10B). An anterolateral facet of the parietal accepts a posterior triangular process of the frontal, forming a scarf joint and giving the parietal an anteriorly tapered shape. A shelf runs longitudinally along the parietal, accepting overlapping processes from both the postfrontal and tabular. Posteriorly the parietal has a straight edge that borders the postparietal. A flange on the underside of the parietal is continuous with the flange of the frontal, extending from the posterior margin of the orbit to the midpoint of the parietal, where it meets its counterpart medially. The suture between the parietals is tight, and there is no evidence of a pineal foramen.

There is a large depression bordered by the descending flanges of the frontals and parietals, similar to that seen in *Rhynchonkos* and *Aletrimyti*, interpreted as a reflection of the dorsal extent of the brain. It was presumably heart-shaped, with a double arched anterior, tapering midway to a point posteriorly.

The postparietal is wide and slightly posteriorly rounded. It has been shifted ventrally, leaving a space between the parietal and postparietal. It has a straight edge anteriorly where it articulated with the parietal. A narrow facet underlaps the parietal and tabular. A small semi-circular notch located at the posterior of the midline suture of the postparietals dorsally exposes the supraoccipital (Fig 10B). The posterior margin of the postparietal meets the exoccipital, overlapping the medial margin of the opisthotic.

The tabular is much smaller than the orbit, is circular, and sits atop facets in the parietal anteromedially and postparietal posteromedially (Fig 10B and 10D). Anteriorly it has a flattened edge that abuts the postfrontal. Two circular fragments, similar in size to that of the tabular, are preserved ventral to the tabular. One is lateral to the quadrate, covering it slightly, and the other is rotated 90 degrees, its lateral face dorsoventrally oriented, located between the quadrate and postorbital. The identity of these fragments is unknown, and they may represent fragments of a larger tabular, or may be part of the quadratojugal or squamosal, which are otherwise absent.

#### Circumorbitals

A ridge of raised bone borders the orbit. The large, circular orbits each occupy approximately 20% of the area of the skull (Fig 10D).

The lacrimal forms the anterior margin of the orbit. It is a broad rectangular element with a slight waisting at the midpoint (Fig 10D). The broad anterior of the lacrimal reaches the septomaxilla and external naris at the anteroventral margin, and a large anterodorsal flange precludes the prefrontal from the external naris. This flange articulates with the nasal, and both the nasal and prefrontal underlap the lacrimal, nearly reaching its midpoint. Ventrally a small flange extends from the maxilla to overlap the lacrimal. A posteromedial flange of the lacrimal contributes to the antorbital wall. This flange reaches the palatine ventrally, and overlaps a similar flange from the prefrontal. No foramina are preserved on this flange; however, CT data reveals a rostrally oriented foramen that bifurcates just anterior to the antorbital wall. These branches are interpreted as the dorsal and ventral openings for the nasolacrimal canal. Medially the lacrimal is smooth and concave. A medially directed shelf extends from the ventral margin, overlapping the maxilla.

The prefrontal is long, extending from the midpoint of the nasal to the dorsal orbital midpoint (Fig 10B). A facet on the prefrontal accepts an overlapping flange of the lacrimal that laterally covers nearly half of the prefrontal. A thin process extends posteriorly to the postfrontal, forming a scarf joint and the anterodorsal margin of the orbit. The prefrontal contributes to the antorbital wall via a descending flange that is overlapped by the flange of the lacrimal. Anterior to this the prefrontal is smooth and medially concave, forming a sinus with the lacrimal for the vomeronasal organ and nasal capsule. Anterodorsally the prefrontal abuts the nasal, and posterodorsally the prefrontal overlaps a facet on the frontal.

The postfrontal is a large, triangular element that forms the posterodorsal margin of the orbit, its anterior point meeting the prefrontal at the orbital dorsal midpoint (Fig 10B). Its straight posterior margin articulates with the tabular posterodorsally to form a butt joint. Anteriorly a long process from the postfrontal nearly reaches the anterior margin of the orbit, underlapping the prefrontal in a scarf joint. Ventrally the postfrontal is slightly overlapped by the postorbital at a small notch.

Ventral to the postfrontal, the postorbital forms the posterior margin of the orbit. It is a roughly trapezoidal element, slightly larger than the tabular, with a slightly rounded posterior end (Fig 10D). Dorsally it sits within a notch on the postfrontal. The raised ridge of bone surrounding the orbit is most pronounced on the postorbital. At the anteroventral margin is a small notch that accepts the jugal. Posteriorly the postorbital borders the ventral cheek emargination.

The jugal is a thin element that forms the posteroventral margin of the orbit (Fig 10D). Anteroventrally it sits within a notch in the maxilla, and posterodorsally within a notch in the postorbital. It is very narrow, less than half the width of the postorbital, and is anteriorly concave. Presumably the jugal formed the anterior border for the ventral cheek emargination.

**Fig 11:**
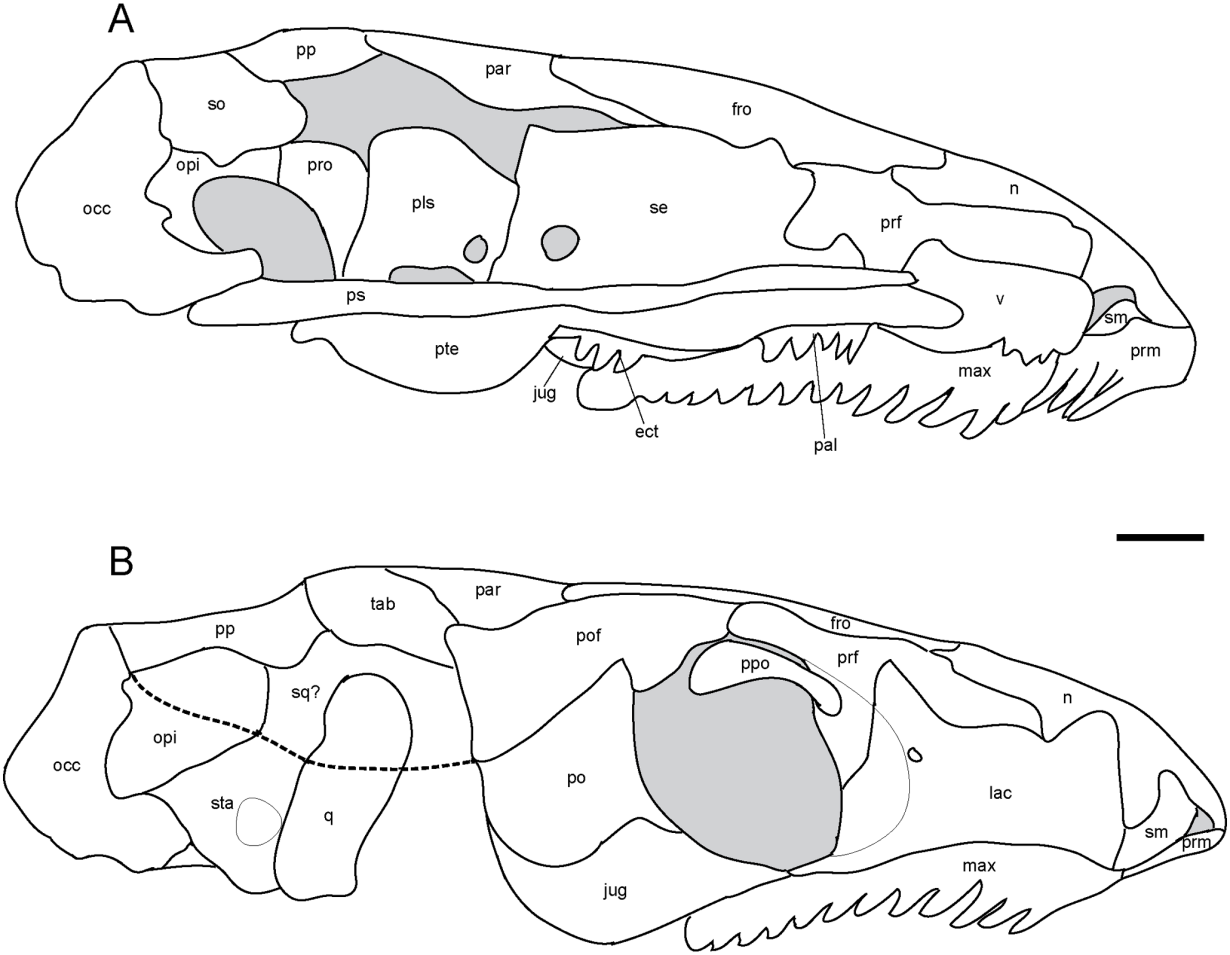
Interpretive drawings of *Dvellecanus carrolli*, gen. et sp. nov. (UCMP 202940). **A**, medial view; **B**, right lateral view. Lower jaw has been removed to better reveal palate and cheek elements. Hashed line indicates presumed ventral border of the squamosal. Scale bar equals 1mm. Abbreviations: **ect**, ectopterygoid; **fro**, frontal; **jug**, jugal; **lac**, lacrimal; **mab**, ‘median anterior braincase bone’; **max**, maxilla; **n**, nasal; **occ**, occipital complex; **opi**, opisthotic; **pal**, palatine; **par**, parietal; **pls**, pleurosphenoid; **po**, postorbital; **pof**, postfrontal; **pp**, postparietal; **ppo**, palpebral ossification; **prf**, prefrontal; **prm**, premaxilla; **pro**, prootic; **ps**, parasphenoid; **pte**, pterygoid; **q**, quadrate; **se**, sphenethmoid; **sm**, septomaxilla; **so**, supraoccipital; **sq**, squamosal; **sta**, stapes; **tab**, tabular; **v**, vomer.

Posterior to the postorbital is the ventral cheek emargination (Figs 10D and 11B). It is large, although its size is exaggerated by the missing squamosal. It is anteriorly bordered by the postorbital, anteroventrally by the jugal, anterodorsally by the postfrontal, dorsally by the tabular, and posteriorly by the quadrate. Two large bone fragments are located within the gap: one at the ventral margin and another at the posteroventral margin (Fig 10D). These fragments may represent the missing quadratojugal or squamosal. The cheek emargination is medially bordered by the quadrate ramus of the pterygoid, and was likely a space for the adductor musculature.

The maxilla is an elongate, slender element that spans from the midpoint of the external naris to the posterior edge of the orbit (Fig 10D). It is ventrally concave, bowing dorsally into the lacrimal. There are approximately 13 peg-like, recurved, non-pedicellate teeth, where the anterior four teeth are the largest, slightly larger than those on the premaxilla, and gradually shorten posteriorly. At the anterior margin of the orbit a small facet is present on the maxilla, extending from the base of the lacrimal to the midpoint of the orbit. Posterior to this is a second facet that accepts the suborbital portion of the jugal. The suborbital process of the lacrimal would have extended to that of the jugal, and would have excluded the maxilla from contributing to the margins of the orbit. Medially a shelf extends from the maxilla to meet the palatine and ectopterygoid, and continues anteriorly to form the lateral border of the choana. A small articulation joins the maxilla and premaxilla ventral to the naris.

A palpebral ossification is present along the dorsal margin of the orbit. It is teardrop-shaped, with its posterior end much broader than the anterior end (Fig 10D).

#### Palate

The wide vomer spans laterally from the midline to the maxilla, unlike that of *Aletrimyti*, and leaves only a small space for the choana in *Dvellecanus* (Fig 3C). It meets the premaxilla anteriorly and a small anteromedial process extends to fit in a notch on the premaxilla at the midline. Laterally the vomer has only a small connection with the maxilla, the rest of the lateral edge forms the medial margin of the choana. Posterolaterally the vomer tightly sutures to the palatine. Posteriorly the vomer overlaps the anterior-most tip of the pterygoid. The vomers do not meet medially, although this is likely taphonomic. The vomers are dorsally concave, with tall dorsoventrally oriented medial walls forming an internasal septum, and slightly upturned lateral walls. Two teeth are preserved on the right vomer, anteromedial to those on the palatine.

The palatine is similar in length to the vomer, but not as wide (Fig 3C). A U-shaped notch at the anterior end of the palatine bordered laterally by the maxilla and medially by the vomer forms the posterior margin of the ovoid choana. The anteromedial margin of the palatine is tightly sutured to the vomer, the medial edge to the pterygoid, and the posterior edge to the ectopterygoid. A medially directed shelf extends from the maxilla to suture to the lateral edge of the palatine. Ventrally, five teeth are preserved on the palatine, the anterior two mediolateral to one another, and the next three anteroposteriorly directed and continuous with those on the vomer.

The ectopterygoid is similar in size to the palatine. Its lateral edge is straight and tightly articulates with the maxilla (Fig 3C). The medial and posterior edges are bordered by the pterygoid, and the posteromedial corner of the ectopterygoid sits within a small notch in the ptergyoid. Anteriorly a small projection of the ectopterygoid fits within a notch walled by the lateral palatine and the medial pterygoid. Three teeth are preserved on the ectopterygoid, in line with those on the palatine.

The pterygoid is the largest element in the palate, spanning from the midpoint of the vomer anteriorly to past the midpoint of the basal plate of the parasphenoid posteriorly (Fig 3C). The palatine process is long, with a pointed anterior end resting ventral to the vomer. The median edge of the palatine process forms the lateral wall of the interpterygoid vacuity, which is narrow, approximately half the width of the palatine process. The pterygoid tightly sutures to the vomer anterolaterally, the palatine laterally, and the ectopterygoid posterolaterally. A U-shaped notch between the intersection of the palatine process and the transverse process accepts the ectopterygoid. The pterygoid is preserved out of articulation with the basipterygoid process of the parasphenoid, shifted slightly laterally. It is likely that the paired pterygoids would have met anteriorly. The quadrate ramus is tall, flat, and medially convex as it spans from the ectopterygoid to the medial margin of the quadrate, nearly completely covering it, except for the posterior-most margin that leaves a small posterior exposure of the quadrate for the stapes. Where the pterygoid meets the palatine process the pterygoid is ventrally concave. Dorsomedial to the palatine process is the epipterygoid, which is no longer in articulation with the pterygoid. No dentition is observed on the pterygoid.

The epipterygoid is poorly preserved. All that remains is a small, circular patch of bone articulating with the basipterygoid process (Fig 3C). It is half the height of the quadrate ramus. A lateral space along the quadrate ramus, between the quadrate and the remains of the epipterygoid, may have been occupied by the complete epipterygoid.

The quadrate is a robust, triangular bone, with points oriented dorsally, laterally, and medially (Fig 5C). It is concave anteriorly, and slightly convex ventrally where it rests atop the articular in a saddle joint (Fig 5F). Medially it is tightly sutured to the quadrate ramus of the pterygoid, and is completely obscured from medial view save for a small posteromedial margin. Dorsally the quadrate reaches the tabular, and it is laterally exposed. At its posteromedial edge it contacts the columella of the stapes. Anteriorly the quadrate forms the posterior border of the ventral cheek emargination.

#### Braincase and Occiput

**Fig 12:**
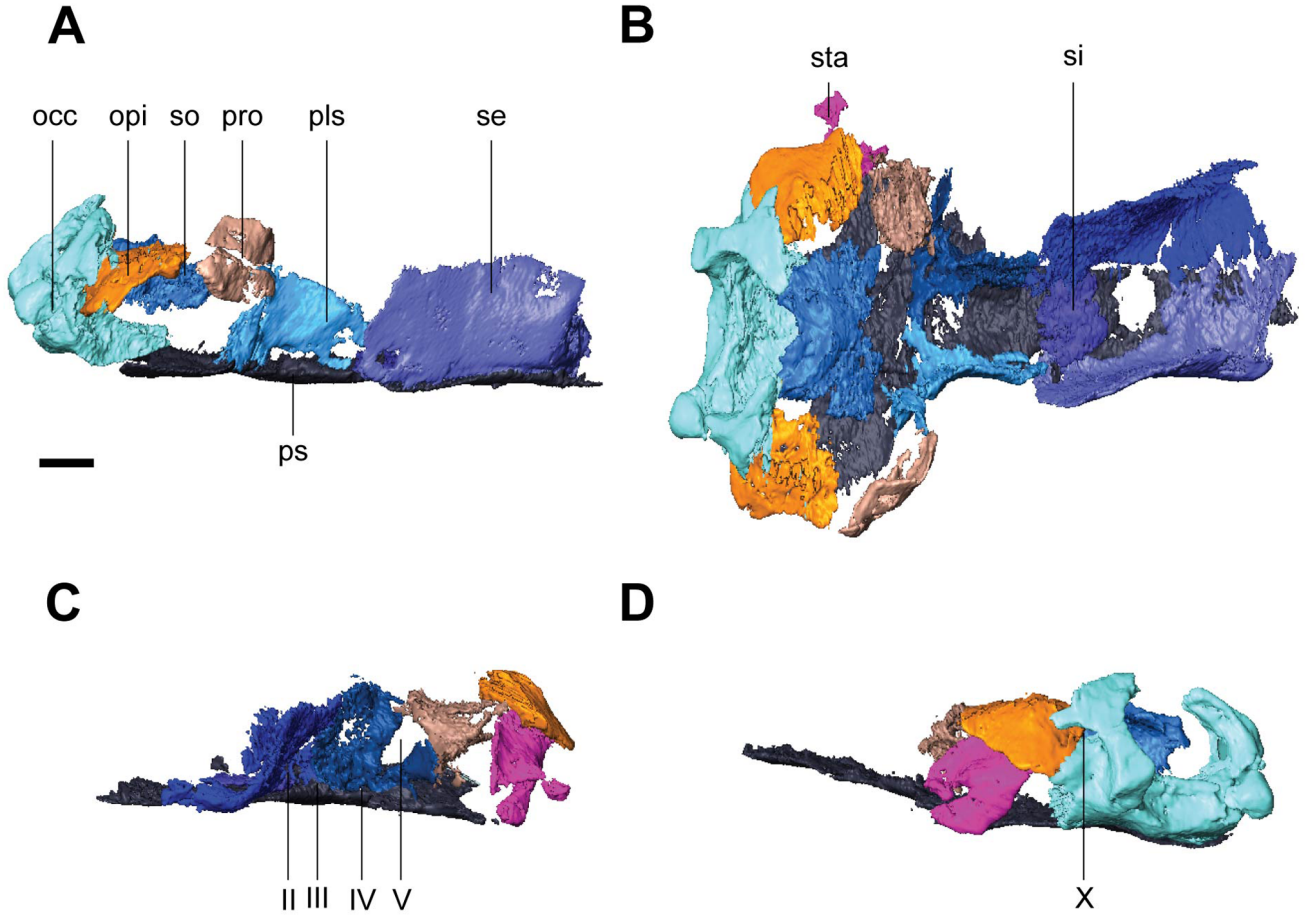
Isolated braincase elements of *Dvellecanus carrolli*, gen. et sp. nov. (UCMP 202940). Shown in right lateral (**A**), dorsal (**B**), left anterolateral oblique (**C**), and left posterolateral oblique (**D**) views. Surrounding elements have been removed to expose braincase. Scale bars equal 1mm. Abbreviations: **occ**, occipital complex; **opi**, opisthotic; **pls**, pleurosphenoid; **pro**, prootic; **ps**, parasphenoid; **se**, sphenethmoid; **si**, ossification of the subiculum infundibulum; **so**, supraoccipital; **sta**, stapes; **II**, optic nerve foramen; **III**, oculomotor nerve foramen; **IV**, trochlear nerve foramen; **V**, fenestra prootica; **X**, foramen for the vagus nerve and jugular vein.

The braincase is robust, spanning from the anterior edge of the frontals to the posterior edge of the parietals (Figs 11A and 12A). Dorsally it reaches the dermatocranium, and maintains its height throughout, with a medial pinching at the pleurosphenoids.

The parasphenoid forms the floor of the braincase. The cultriform process is long and broad, extending from midway through the occiput to just ahead of the anterior margin of the level of the orbit (Fig 3C). The cultriform process is slightly concave dorsally where it is flanked by the sphenethmoids, and the anterior tip is somewhat pointed and extends anteriorly past the sphenethmoids. The cultriform process is horizontally level with the elements of the palate. At its base where it meets the basal plate, only a partial fragment of the right basipterygoid process remains. Anterodorsal to this is a shallow recess for the hypophyseal fossa, bordered laterally by the pleurosphenoids (Fig 11B). The basal plate was large and roughly triangular, but is poorly preserved. It is rectangular in shape, spanning from midway through the parietals to the posterior edge of the postparietals and laterally falls short of the stapes. The basal plate would likely have overlapped the anterior half of the occiput.

The sphenethmoids are large and rectangular, slightly bulging laterally as they rise dorsally from the parasphenoid to the skull roof (Fig 12A). They nearly span the entire length of the cultriform process. Anteriorly, the sphenethmoids meet medially with a clear suture between them, and a small dorsal projection that rises to half the height of the sphenethmoids. This projection is formed equally by both sides, although the right is poorly preserved, and presumably would have closed the anteroventral margin of the braincase. The optic foramen is wholly housed in the posteroventral corner of each sphenethmoid (Fig 11C). The sphenethmoids tightly contact the pleurosphenoids posteriorly, and form the anterior border to the oculomotor foramen (Fig 11C). The descending flange from the frontal overlaps the sphenethmoid laterally, sitting in an embayment and occupying slightly over half the length of the orbit. The descending flange from the parietal sits in a notch in the sphenethmoid and extends to the posterior edge of the sphenethmoid.

Medial to the sphenethmoids is an ossification of the subiculum infundibuli (Fig 11B). It is a narrow piece of bone that provides the anterior border to the hypophyseal fossa, and the posterior border to an extension of the ventral brain between it and the anterior half of the sphenethmoids. It medially connects the sphenethmoids at their posterior end.

The pleurosphenoids directly articulate to the sphenethmoids (Fig 12A). The pleurosphenoids are slightly more than half the length of the sphenethmoids, and are of a similar height, but they fail to reach the skull roof. The pleurosphenoids are more narrowly-spaced than the sphenethmoids, however, in constrast to the state observed in *Rhynchonkos*, *Aletrimyti*, and some other recumbirostrans [16, 18]. At the posterior of the pleurosphenoids is a laterally oriented process that extends posterior to the basipterygoid processes. Where the process turns laterally there is a gap in the pleurosphenoids, interpreted as the fenestra prootica, allowing for the passage of the trigeminal nerve (Fig 11C). The pleurosphenoids provide the lateral and posterior walls to the hypophyseal fossa. The oculomotor foramen is present along the sphenethmoid-pleurosphenoid contact at approximately the level of the optic foramen (Fig 11C), with the pleurosphenoid contributing the dorsal, ventral, and posterior border of the foramen. Posterior to this is the trochlear foramen (Figs 11C and 12A), located dorsal to the basipterygoid processes, ventrally bordered by the parasphenoid.

The occiput is composed of a co-ossified basioccipital and exoccipitals, and a supraoccipital (Figs 10B and 12A). The basioccipital is a broad element, slightly wider than the basipterygoid processes of the parasphenoid, with an oval-shaped interior that houses the foramen magnum. Its anterior half is ventrally bordered by the parasphenoid and is ventrally sloping, with a short margin that extends posteriorly past the basal plate of the parasphenoid. The occipital cotyle is anteriorly concave. A pair of well-developed knob-like occipital condyles extends posteriorly, flanking the occipital cotyle and the foramen magnum. Anterodorsal to these condyles is a pair of large foramina between the occipital complex and the opisthotic, interpreted as the jugular foramen (Fig 11D). The exoccipitals are broadly sutured to the opisthotics, with a small point of contact to the postparietals at the anterodorsal-most margin of the exoccipitals. The supraoccipital is short and ventrally convex. Its anterior is found midway through the postparietals and is separated from the pleurosphenoids by the dorsal sinus. The supraoccipital has been shifted ventrally and is out of contact with the exoccipitals; presumably it would have medially contacted both exoccipitals. Seen dorsally it is rectangular in shape, with short lateral processes. Seen medially there is a C-shaped groove that opens laterally on either side of the supraoccipital. This groove is continuous with that of the otic capsules and likely contributed to housing the horizontal semicircular canals.

#### Otic Capsules

**Fig 13:**
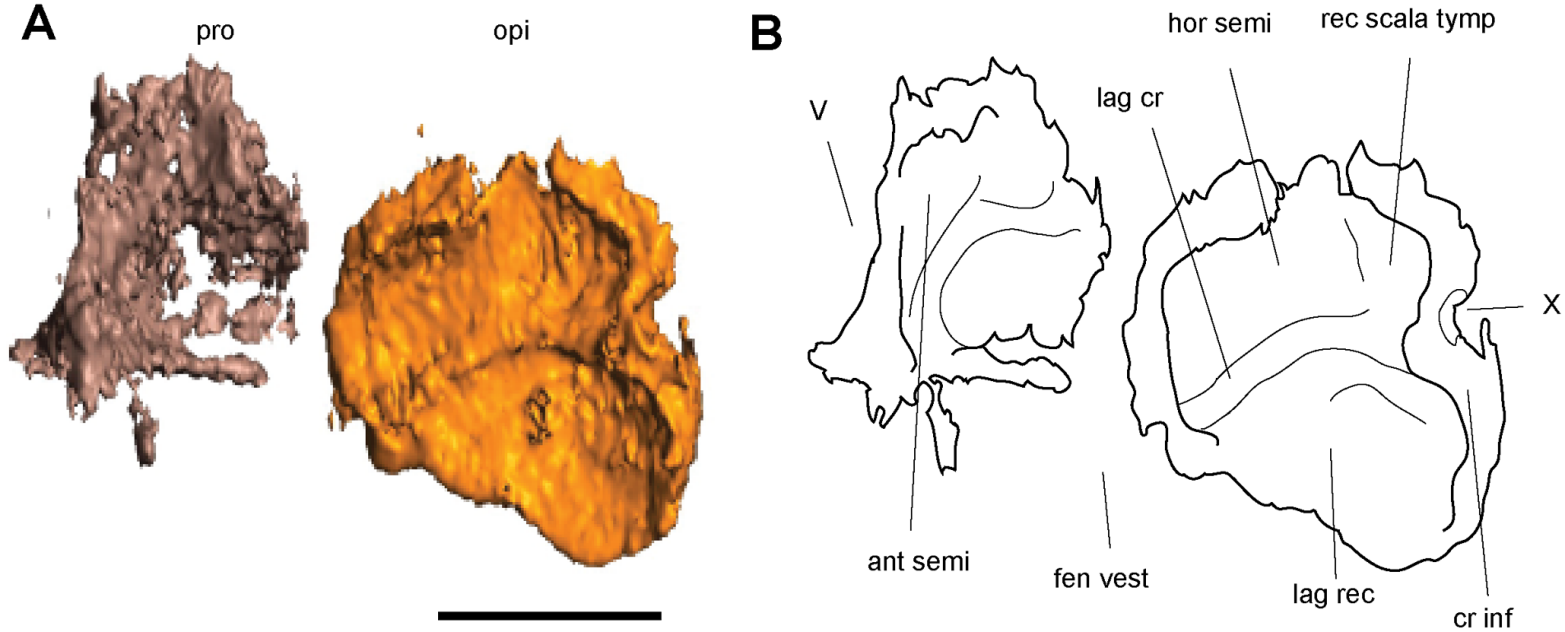
Isolated elements of the right otic capsule of *Dvellecanus carrolli*, *gen*. *et sp*. *nov*. (UCMP 202940). Scan (**A**) and interpretive line drawing (**B**) in ventromedial oblique view, with the anterior oriented to the left. Surrounding braincase elements have been removed to expose the otic capsule. Scale bar equals 1mm. Abbreviations: **ant semi**, groove for the anterior semicircular canal; **cr inf**, crista interfenestralis; **fen vest**, fenestra vestibuli; **hor semi**, groove for the horizontal semicircular canal; **lag cr**, lagenar crest; **lag rec**, lagenar recess; **opi**, opisthotic; **pro**, prootic; **rec scala tymp**, recessus scala tympani; **V**, fenestra prootica; **X**, foramen for the vagus nerve and jugular vein.

The otic capsules are composed of a distinct anterior prootic and posterior opisthotic, with the opisthotic slightly larger than the prootic (Fig 13A and 13B). The otic capsules are large, spanning the length of the basal plate of the parasphenoid and together occupying over two-thirds the width of the skull. Only the opisthotic reaches the dermatocranium; the prootic is separated from the skull roof by the dorsal sinus. The only point of articulation between the otic capsule and the parasphenoid is a small contact at the anteroventral margin of the otic capsule. Posteriorly the otic capsule is firmly sutured to the occipital complex. The otic capsule is arched where it encloses the fenestra vestibuli. It has a ventrally deflected anterior surface that reaches the parasphenoid lateral to the pleurosphenoid and forms the lateral margin of the fenestra prootica. On the dorsal margin of the otic capsules is a medially oriented shelf that reaches the lateral processes of the supraoccipital. Ventral to this shelf is a space for the horizontal semicircular canal that spans the length of the otic capsules, and ventral to the semicircular canal is the fenestra vestibuli. In dorsal view the otic capsules are medially flat and laterally rounded.

The prootic has a flattened anterior surface, rounding posteriorly as it covers the fenestra vestibuli (Fig 13A and 13B). There is a small point of contact between the prootic and the parasphenoid at the anterolateral margin. As the prootic rises from the parasphenoid, it forms the lateral wall to the fenestra prootica. At its anteromedial margin the prootic contacts the pleurosphenoid. The fenestra vestibuli occupies the posterior half of the prootic. Slightly less than half of the posterior of the prootic is occupied by the groove for the horizontal semicircular canal. Medially the prootic is flat, except at the posteromedial margin where it is incised laterally. Here the supraoccipital sutures to the dorsal shelf of the prootic. The prootic was likely completely obscured from lateral view by the tabular and squamosal. At the anterodorsal margin of the prootic a dorsoventrally-oriented groove is preserved, interpreted as for the anterior semicircular canal.

The opisthotic meets the prootic in a straight articulation. Due to its greater size, the opisthotic contributes a greater portion to the fenestra vestibuli and to the groove for the horizontal semicircular canal (Fig 13A and 13B). Like the prootic it is rounded where it covers the fenestra vestibuli and has a sharp ventrally sloped posterior margin that sutures to the occipital complex. Posteromedially between the opisthotic and the occipital complex is a large foramen, likely the jugular foramen. The opisthotic reaches the dermatocranium and tightly contacts the postparietals dorsally. Laterally and posteriorly the opisthotic is exposed in view. Medially the opisthotic is flat with a slight incision at the anteromedial margin, and is sutured to the supraoccipital for the length of the dorsal shelf of the opisthotic.

The stapes is ovoid and medially concave. It fills the margins of the fenestra vestibuli, leaving little space between it and the otic capsule. The columella is relatively long, robust, and reaches the quadrate. The base of the columella is pierced by the stapedial foramen.

#### Lower Jaw

The lower jaw is composed of a dentary, prearticular, articular, angular, surangular, coronoid, splenial, and post-splenial (Fig 5C and 5F). It extends to the anterior margin of the otic capsules, ending just before the stapes. There is a slight tapering anteriorly towards the symphysis, and it broadens at the coronoid process of the dentary. The posterior of the lower jaw is slightly rounded, and ends abruptly at the articular in a straight edge. The symphysis is solely composed by the dentary. The dentary is long and slender, with a slight tapering anteriorly. The coronoid process is upturned and extends slightly posteriorly past the level of the orbit, ending at the level of the midpoint of the postorbital. There are approximately 17 peg-like teeth on the dentary, with the four posterior-most smaller than the others. The tooth row ends somewhat posterior to the anterior margin of the coronoid.

The coronoid is a broad element, with its anterior margin at the level of the 15^th^ tooth of the dentary and its posterior margin is located midway through the length of the surangular. A few denticles are preserved on the coronoid. There is a large lateral exposure of the coronoid, spanning the dorsal portion of the coronoid process of the dentary and approximately one-third of the anterior-most dorsal portion of the surangular. The splenial is short, albeit broad, its length spanning approximately from the level of the second to eighth tooth and it fails to reach the symphysis. The post-splenial is much longer and narrower than the splenial, beginning at the posterior edge of the splenial, extending below the prearticular, and ending at the level of the anterior margin of the coronoid prominence. The prearticular is located at the midpoint of the dentary. It is long and rod-like, spanning from the level of the fifth tooth to just past the posterior edge of the coronoid. The prearticular is a broad element that is over half the width of the dentary. The angular is of similar length, beginning posterior to the splenial and spanning the rest of the length of the jaw, where it ends in a straight edge. It has a large lateral exposure that tapers anteriorly and is almost as broad as the dentary posteriorly. The angular forms the posterior half of the ventral margin of the lower jaw. The surangular borders the angular dorsally and rises to the coronoid process, after which it gradually tapers, giving the lower jaw a triangular shape in lateral view. Like the angular, the surangular ends in a flat edge. Both are overlapped anteriorly by the coronoid process of the dentary. The articular is a short, robust element, its anterior margin appearing slightly posteriorly to the coronoid. A small posterior notch accepts the trochlear quadrate. It is completely obscured from lateral view by the angular and surangular, with no retroarticular process.

The Meckelian fossa is long and triangular. Anteriorly it tapers to a point, and gradually broadens towards the posterior jaw. It is dorsally bordered by a medially-oriented shelf below the tooth row on the dentary and by the coronoid. The dentary also provides the lateral and ventral borders between the Meckelian fossa’s anterior margin at the level of the second tooth. The angular replaces the borders formed by the dentary after the eighth tooth until the posterior margin of the Meckelian fossa at the articular. The prearticular provides the medial wall of the Meckelian fossa for nearly the entire medial margin; the splenial only forms the medial wall between teeth two through eight. At the level of the coronoid prominence of the dentary the Meckelian fossa broadens to fill the posterior jaw margins, where it is laterally walled by the surangular and angular. The only visible foramen for the Meckelian fossa is present between the coronoid prominence of the dentary and the angular.

## Discussion

### Morphological diversity within ‘*Rhynchonkos*’ and patterns of fossoriality within recumbirostrans

Superficially, all three specimens previously attributed to *Rhynchonkos stovalli* have the typical tuditanomorph skull roof pattern (the parietals, frontals, and nasals are of subequal size; the postfrontals and postorbitals are of subequal size, and border on large tabulars) [19] that follows the same interlocking pattern between elements (Figs 1, 7 and 10). More traditional methods of differentiation, such as size comparisons between bones in the skull roof, have led to these specimens being lumped together into a single species; any observed differences were attributed to taphonomy or intraspecific variation. Furthermore, the braincase, lower jaw, and axial skeleton of *Rhynchonkos* were inferred from separate specimens, making the original *Rhynchonkos stovalli* description a composite of multiple specimens.

Considering small differences to be taphonomic can have considerable implications. For example, in *Rhynchonkos*, the ventral margin of the orbit is bordered by the maxilla, with a small anterior participation from the lacrimal and a small posterior participation by the jugal. *Aletrimyti* shows a similar condition, albeit with a greater participation from the lacrimal. The suborbital in *Dvellecanus* is equally bordered by the lacrimal and the jugal; however, the suborbital processes of the lacrimal and jugal have not been preserved, giving the orbit the appearance of being ventrally bordered by the maxilla. Similarly, the external nares of *Rhynchonkos* and *Dvellecanus* are posteriorly bordered by the lacrimal, whereas in *Aletrimyti* the lacrimal is excluded from the naris by a descending process of the nasal and a small process from the septomaxilla. The cheek region of *Rhynchonkos* is fully encased in bone, with the squamosal meeting the jugal to cover the quadrate. In *Aletrimyti*, the squamosal fails to meet the jugal, leaving a gap in the lower cheek. Reconstructing the maxilla as the ventral border for the orbit, or the squamosal to cover the cheek, renders all three skulls similar.

**Fig 14:**
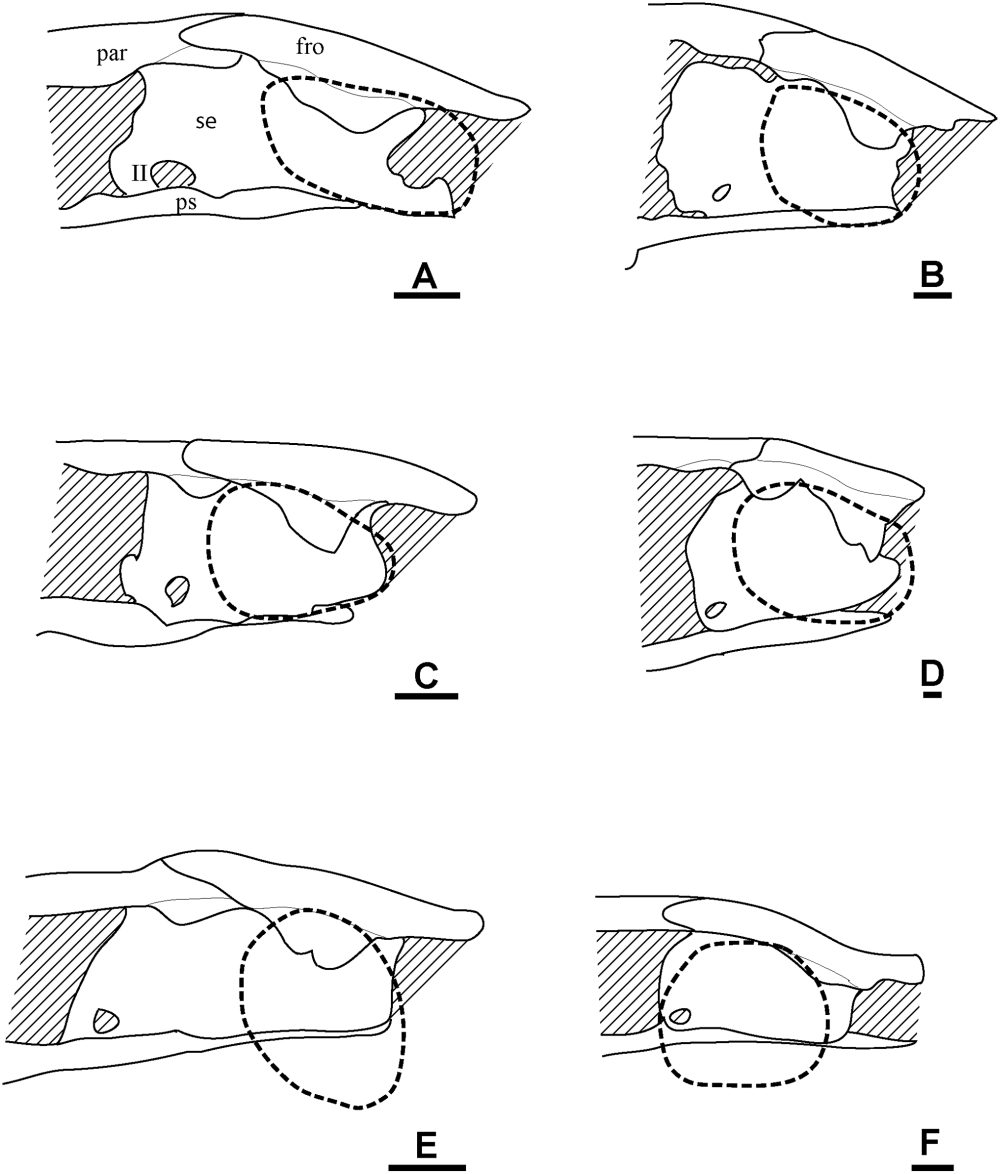
Reconstructions of the sphenethmoid and the descending flange of the frontal and parietal. Specimens previously attributed to *Rhynchonkos* (**A**, **C**, **E**) and select microsaurs (**B**, **D**, **F**) in right lateral view. **A**, *Aletrimyti gaskillae*, gen. et sp. nov. (FM-UR 1040); **B**, *Nannaroter mckinziei* (OMNH 73107); **C**, *Rhynchonkos stovalli* (FM-UR 1039); **D**, *Micraroter erythrogeios* (FM-UR 2311); **E**, *Dvellecanus carrolli*, gen. et sp. nov. (UCMP 202940); **F**, *Huskerpeton englehorni* (UNSM 32144). Dashed line indicates the bony margins of the orbit. Scale bars equal 1mm. Abbreviations: **fro**, frontal; **par**, parietal; **ps**, parasphenoid; **se**, sphenethmoid; **II**, optic nerve foramen.

It is only when considering the internal morphology that the largest differences between specimens are observed. Possibly the greatest difference in internal morphology is seen in *Aletrimyti*, where the elements of the braincase and palate articulate with those of the dermatocranium. It is a common feature amongst recumbirostrans for the sphenethmoid to reach the skull roof (Fig 14). In *Aletrimyti*, not only does the sphenethmoid brace against the frontals, but the pleurosphenoids articulate with the parietals, the vomers with the nasals, and the ‘median anterior braincase bone’ with the frontals (Fig 8A). *Rhynchonkos* shows a lesser degree of dorsoventral bracing, with only the sphenethmoid and epipterygoids reaching the skull roof, while in *Dvellecanus* only the frontals contact the sphenethmoids. Likewise, *Dvellecanus* shows an enlarged dorsal sinus, similar to the condition seen in *Pelodosotis*, beginning between the sphenethmoids and the parietals and spanning to the supraoccipital (Fig 12A). *Aletrimyti* shows a much smaller sinus that occupies the area dorsal to the supraoccipital and otic capsules (Fig 8A), while the only evidence of a sinus in *Rhynchonkos* is a narrow gap between the pleurosphenoids and parietals (Fig 4A). The shape of the occiput of *Rhynchonkos* differs from both *Aletrimyti* and *Dvellecanus*. Whereas *Rhynchonkos* has a somewhat flattened set of occipital condyles, *Aletrimyti* and *Dvellecanus* have a much more convex occiput with more pronounced exoccipitals that form the knob-like condyles that flank the cotyle of the basioccipital.

Although the possibility exists that the characters we identify here represent ontogenetic variation within a single species rather than interspecific variation, we consider this unlikely on the basis of several lines of evidence. Although gross morphometric measures, such as basal skull length and relative orbital size, may vary allometrically, a number of the characteristics we recognize here involve novel ossifications or co-ossifications of braincase elements (fusion of the basioccipital and exoccipital in *Dvellecanus*, presence of an ossified columella ethmoidalis in *Aletrimyti*, absence of ossified contact between the sphenethmoid and pleurosphenoid in *Rhynchonkos*), as well as differences in presence and absence of braincase structures that are unlikely to be ontogenetically variable (e.g. presence of a dorsal sinus between the supraoccipital and posterior skull roof in *Aletrimyti and Dvellecanus*, short, squared-off ascending processes of the supraoccipital in *Dvellecanus*). Moreover, all three forms exhibit tight sutures between ossifications of the dermatocranium, well-ossified septomaxillae and otic capsules, and prominent palatal teeth, indicating that all three forms likely represent adult individuals. No credible ontogenetic series encompassing two or more of these morphotypes can be constructed that would not require substantial reorganization of the skull and braincase at a relatively late stage of development. We reject the possibility that these morphotypes represent juveniles of other larger recumbirostran taxa, such as the ostodolepid *Micraroter erythrogeios*, on similar grounds.

Differences in morphology between *Rhynchonkos*, *Aletrimyti*, and *Dvellcanus* may reflect differences in burrowing performance between these three taxa. The greater degree of bracing seen in *Aletrimyti* is likely a more derived feature for head-first burrowing, reducing the compression forces experienced along the skull and protecting the brain and sensory organs from crushing [26]. The ‘cranial box’ morphology is also seen in amphisbaenids [26], as well as in burrowing snakes [27] and burrowing skinks [28]. The posterior dorsal sinus may be the result of increasing the surface area at the posterior of the skull for epaxial muscle insertion [16,18], thereby increasing the amount of substrate the animal is capable of displacing during the digging stroke [26]. The greater rounding of the occipital condyle-cotyle complex and lowering of the atlas neural spine may also be derived features for head-first burrowing, restricting lateral movements of the skull while leaving it free to move dorsoventrally, reducing the risk of injury during both ramming and digging strokes.

In head-first burrowing the cross-sectional area of the skull is directly proportional to the amount of resistance encountered during the ramming stroke, and to the force required to propel the animal forward through the substrate [26]. The emargination of the cheek region may function as a means of reducing the cross-sectional area of the skull (Table 1) by increasing the internal space for the adductor musculature, and decreasing the amount of muscle outside the skull. Ventral emargination of the cheek region is common among recumbirostrans and closely-related ‘microsaurs,’ and has been recognized in the hapsidopareiontids *Hapsidopareion* and *Llistrofus* [19], and in the ostodolepids *Pelodosotis*, *Micraroter* [19], *Tambaroter* [29], and *Nannaroter* (where it is not preserved in its entirety but its presence is inferred based on the shape of the jugal) [17]. In *Aletrimyti* and *Dvellecanus*, a cheek emargination has been observed as a space posterior to the jugal (Figs 7B and 10B), suggesting that these taxa may have been subject to greater substrate constraint, and thus more fossorial, than *Rhynchonkos*.

**Table 1:** Skull measurements (in millimeters) of *Rhynchonkos stovalli* (FM-UR 1039), *Aletrimyti gaskillae*, *gen*. *et sp*. *nov*. (FM-UR 1040), *Dvellecanus carrolli*, *gen*. *et sp*. *nov*. (UCMP 202940), and select microsaurs (*Nannaroter mckinziei* (OMNH 73107), *Microroter erythrogeios* (FM-UR 2311), and *Huskerpeton englehorni* (UNSM 32144)).

	*Rhynchonkos*	*Aletrimyti*	*Dvellecanus*
Skull height	4.42	4.96	4.73
Skull length	13.77	19.5	14.15
Skull width	10.89	10.94	11.38
Skull length/width ratio	1.26	1.78	1.24
Antorbital region	4.61	6.37	4.61
Orbit	3.28	3.32	2.28
Postorbital region	5.88	9.81	7.26
Postorbital/Antorbital ratio	1.28	1.54	1.57
Cross-sectional area (approx.)	45.28	51.12	52.01
	*Nannaroter*	*Micraroter*	*Huskerpeton*
Skull height	9.06	17.51	5.71
Skull length	17.05	42.38	19.37
Skull width	12.93	26.84	16.04
Skull length/width ratio	1.32	1.58	1.21
Antorbital region	6.62	14.14	5.56
Orbit	4.07	8.75	3.62
Postorbital region	6.38	19.49	10.13
Postorbital/Antorbital ratio	0.96	1.38	1.82
Cross-sectional area (approx.)	93.61	320.15	92.16

Furthermore, feeding in burrows requires the animal to capture and process prey within the confined space of a burrow, both restricting maximum gape and reducing the overall mechanical advantage of the jaw closure mechanism. In amphisbaenians, this is compensated for with greatly enlarged mandibular adductor muscles, which insert onto a high, well-developed coronoid process of the dentary [30]. An expanded coronoid process with a lateral exposure of the coronoid in ostodolepids [19] and the taxa described here may have served a similar function. Mechanical advantage during jaw depression is also reduced, and, in amphisbaenians, the depressor mandibulae are expanded in compensation and insert at an enlarged retroarticular process. An elongate retroarticular process is seen in *Aletrimyti* and, to a lesser extent, in *Rhynchonkos*.

Another consequence of feeding within the confined space of a burrow is that opening of the jaw against resistant substrate imposes substantial forces on the ventral margin of the mandibular ramus. Amphisbaenians compensate for this by expanding the dentary and reducing the size and number of the other elements of the lower jaw, relying on a single element to withstand the force of jaw opening [30]. The reduction of the number of coronoids from two or three in other taxa such as *Huskerpeton* [18] and *Pantylus* [31] to the single coronoid in ostodolepids [19], brachystelechids [16], and two of the taxa described here may be evidence of a general trend towards the reduction of the lower jaw in recumbirostrans convergent with that seen in amphisbaenids. Expansion of the dentary into the dominant mandibular element and continued reduction of the Meckelian fossa in the brachystelechid *Carrolla* [16] may be further evidence of this trend. This coronoid is a long element covered in denticles, and is the only coronoid in the lower jaw. *Pelodosotis* shows a similar lateral exposure of the coronoid [19]. The posterior of the lower jaw of *Nannaroter* is not wholly preserved; what remains of the coronoid is a single long, toothed bone that rises to be level with the coronoid process of the dentary. Likewise, the coronoid of *Micraroter* is a single long, toothed element that remains level with the dorsal margin of the coronoid process of the dentary.

Taken together, these features imply that *Aletrimyti* and, to a lesser extent *Dvellecanus*, were more derived for a burrowing lifestyle than *Rhynchonkos*, and that its different morphology may have had functional implications that allowed it to explore new niches. Furthermore, this implies that not only were recumbirostrans exploring head-first burrowing and fossoriality early on [17,18], but they were showing signs of adaptation and diversification within this highly specialized niche as early as the Lower Permian. With the variety in endocranial features seen in the specimens previously attributed to *Rhynchonkos*, it is possible that other superficially-similar taxa may be functionally different, and recumbirostrans may be more morphologically diverse than previously thought.

### Homology of the recumbirostran supraoccipital

A single median ossification is seen in the synotic tectum of all recumbirostrans [18,19,31] but the homology and morphology of this ossification have remained unclear. Recent authors have argued against homology between this ossification and the supraoccipital bone of amniotes, in part because some lepospondyls (primarily nectrideans [32], but also microbrachomorph microsaurs [13]) and all seymouriamorphs lack such an ossification [33]. Thus, a single median ossification within the syntotic tectum has been added to a long list of hypothesized convergences between recumbirostrans and amniotes.

Ossifications within the synotic tectum are common among early vertebrates. In early stem-tetrapods, the synotic tectum is massively co-ossified with the otic capsules (but is separated from the occipital arch by the otoccipital fissure), as has been described in *Acanthostega* [34]. Distinct supraoccipital ossifications arise in embolomere-grade tetrapods such as *Archeria* [35] and baphetids such as *Kyrinion* [36], but remain separated from the occipital arch by the otoccipital fissure. The otoccipital fissure closes by the origin of the tetrapod crown group, but ossifications of the synotic tectum are not ubiquitously preserved, and are absent in many temnospondyls [37] where the synotic tectum is instead invaded by processes of the exoccipitals, as well as colosteids [38] and seymouriamorphs [33], where the synotic tectum is largely unossified. It is worth noting, however, that the supraoccipital typically ossifies late in ontogeny in comparison with the other bones of the occipital arch [39,40], and absence of an ossification in the synotic tectum may be indicative in at least some cases that the studied material is either paedomorphic or immature. Indeed, Watson [41] reported in passing a possible ossification of the synotic tectum in a large specimen of *Seymouria baylorensis*. Arguments using ancestor state reconstruction of the presence or absence of the supraoccipital should address these ontogenetic and heterochronic effects.

An additional line of evidence that has not been used to address the homology of the recumbirostran supraoccipital is the comparative morphology of this element and supraoccipitals of amniotes and early tetrapods. Previous investigations of the homology of the recumbirostran supraoccipital have generally been limited to the occipital exposure of this bone, and have been unable to access detailed anatomy of this element for comparison with the supraoccipital in amniotes. The high quality 3D data presented here ameliorates this to some degree.

**Fig 15:**
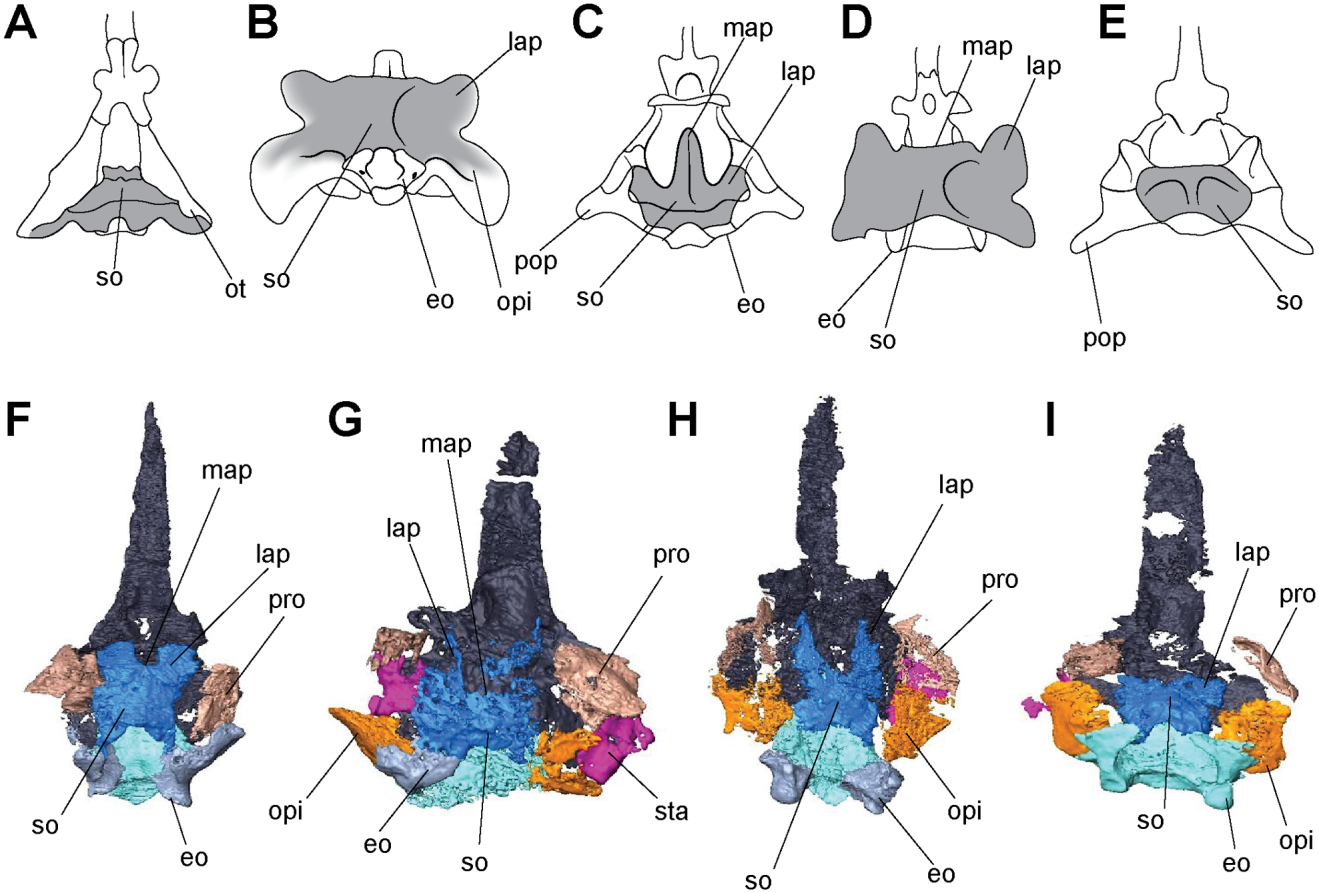
Posterior braincases of selected tetrapods in dorsal view, showing ossifications of the synotic tectum. Interpretive line drawings (**A-E**) and volumizations (**F-I**). **A**, *Limnoscelis palustris*, after [42]; **B**, *Aerosaurus* cf. *A*. *wellesi*, after [43]; **C**, *Captorhinus laticeps*, after [44]; **D**, *Petrolacosaurus kansensis*, after [45]; **E**, *Youngina capensis*, after [46]; **F**, *Huskerpeton englehorni* (UNSM 32144); **G**, *Rhynchonkos stovalli* (FM-UR 1039); **H**, *Aletrimyti* (FM-UR 1040); **I**, *Dvellecanus* (UCMP 202940). Images not to scale. Abbreviations: **eo**, exoccipital; **lap**, lateral ascending process; **map**, median ascending process; **opi**, opisthotic; **ot**, otic; **pop**, posterior process; **pro**, prootic; **so**, supraoccipital; **sta**, stapes.

The supraoccipitals of *Rhynchonkos*, *Aletrimyti*, *Dvellecanus* and *Huskerpeton* [18] exhibit broad winglike processes that extend parasagitally from the main body of the supraoccipital (Fig 15F–15I). These processes roughly follow the parasagittal septum separating the cavum cranium and utricular fossa, and that septum is partially preserved as an ossified crista on the ventral surface of these processes. These processes are separated medially by the posterior fontanelle. A small median process can be observed in *Huskerpeton* (Fig 15F) extending weakly into the posterior fontanelle.

Very similar anatomy of the supraoccipital has been described in amniotes, specifically eureptiles, in which the winglike lateral processes flanking the posterior fontanelle are generally referred to as lateral ascending processes, and the median protuberance is named the median ascending process. This anatomy is consistent throughout eureptiles, appearing in the captorhinid *Captorhinus laticeps* (Fig 15C) [44], and the areoscelid *Petrolacosaurus kansensis* (Fig 15D) [45], with reduced median and ascending processes in the neodiapsid *Youngina* (Fig 15E) [46]. Lateral and median ascending processes are not found in more basal tetrapods [34–36] and are not ubiquitous among amniotes; such processes are not present in the parareptile *Macroleter poezicus* [47], in the diadectamorph *Limnoscelis* (Fig 15A) [42], or in synapsids (Fig 15B) [43].

The precise similarities of the morphology of the recumbirostran supraoccipital and the supraoccipital of eureptiles are difficult to assess within the current consensus of stem- and crown-amniote phylogeny, but the extent of anatomical similarity between these elements offers substantial support to the hypothesis of homology between these elements. Although this homology ultimately must be tested phylogenetically, we suggest that this bone be treated as a homolog of the amniote supraoccipital rather than a neomorph. This approach facilitates comparison of the morphology of the amniote and recumbirostran supraoccipitals, and avoids canonizing phylogenetic results that may be based on incomplete description of recumbirostran morphology.

### Homology of the recumbirostran temporal bone

In the plesiomorphic condition, three bones make up the temporal region of the tetrapod skull roof: the intertemporal, supratemporal, and tabular. Tetrapods demonstrate numerous parallel reductions in the number of bones in the temporal region, with loss of one or more bones in that region characterizing Diadectamorpha+Amniota, Recumbirostra, Adelospondyli, Diplocaulidae, Keraterpetontidae, Dvinosauroidea, Dissorophoidea, Phlegethontiidae, and several other lineages.

In recumbirostrans, the temporal region consists of a single bone. The homologies of this element have been discussed in detail [19,48,49]. Romer [48] argued for homology of the temporal bone with the tabular based on participation of the element in the occipital margin of the skull roof. However, it is worth noting that in some reptiles (e.g. *Captorhinus laticeps*) the supratemporal exhibits an occipital exposure [44], and the tabular is completely lost, and in some reptiles (e.g. *Petrolacosaurus* kansensis) [45] as well as some temnospondyls (e.g. *Acroplous vorax*) [50], an occipital exposure of supratemporal is present lateral to a reduced tabular bone. The homology of this element has non-trivial implications for phylogenetic analysis of recumbirostrans among early tetrapods. In the most complete recent analysis of recumbirostran relationships [11], seven character state codings are dependent on whether this element is interpreted as a supratemporal or tabular. Similar character nonindependence issues appear in other recent character matrices [13,51], with the potential to dramatically mislead phylogenetic analysis of recumbirostran relationships. Ultimately, the homology of this element will need to be reassessed, either by identifying new anatomical evidence in favor of homology statements, or by using methods or approaches that allow phylogeny to be assessed without arbitrarily resolving the homological ambiguity of this element.

### Homology of the ‘median anterior braincase bone’

An ossification medial to the sphenethmoids was noted by Maddin et al. [16] in their redescription of *Carrolla*, which they termed the ‘median anterior braincase bone’ and tentatively attributed as an ossification of the ethmoid region. Similar ossifications are present in *Aletrimyti*, *Rhynchonkos*, and *Huskerpeton*, and appear to represent a neomorphic ventral ossification of the ethmoid region that may fully invade the columella ethmoidalis (as in *Carrolla* and *Aletrimyti*) or be restricted to ventral region of the ethmoid region (as in *Huskerpeton* and *Rhynchonkos*). Some morphological variation of this structure is present; in *Carrolla craddocki*, the median anterior braincase bone is expanded ventrally into a deep dorsoventral keel that vaults the cavum cranii above the palatal surface in a manner reminiscent of the interorbital septum observed in many early amniotes (e.g. *Captorhinus laticeps*, [44]), whereas in most taxa, the main body of this element is relatively low and flat. The dorsal process of this element in *Carrolla* and in *Aletrimyti*, identified here as an ossified columella ethmoidalis, is dorsally expanded and braces against the ventral surface of the nasals, possibly reinforcing the ethmoid region against dorsoventral compression. The columella ethmoidalis seems contiguous with a median lamina of the vomer, which may have contributed to the septum nasalis. The distribution of this element among other recumbirostrans remains unclear; the deep ethmoid region described in *Quasicaecilia texana* may be the same element [52], but it is apparently absent in *Dvellecanus* and is not apparent in transverse micro-CT scans of *Nannaroter mckinziei* [17]. In extant squamates, the morphology of median skull ossifications has a large impact on phylogeny [53]; this may ultimately also be the case in recumbirostrans but until more data becomes available, the homology of this element remains uncertain.

### *Rhynchonkos* and caecilian ancestry

Although a close relationship between *Rhynchonkos* and gymnophionans has been recovered in a number of recent analyses [2,11,12,18] new data from soft tissue and inner ear anatomy [15] and from micro-CT study of the braincase of the Jurassic gymnophionan *Eocaecilia micropodia* [6] have failed to support a *Rhynchonkos*-gymnophionan relationship, and instead have supported placement of a monophyletic Lissamphibia within dissorophoid temnospondyls. Our revision here of the morphology of *Rhynchonkos*, *Aletrimyti*, and *Dvellecanus* may bolster these new results in subsequent analyses. Although a number of characteristics common to *Rhynchonkos* and gymnophionans have been enumerated [8,9], some differences between the description presented by Carroll and Currie [8] and Carroll and Gaskill [19] and the revised descriptions of *Rhynchonkos*, *Aletrimyti*, and *Dvellecanus* presented here necessitate reconsideration of these synamoporphies.

A strong suture between the vomers and cultriform process of the parasphenoid is noted as possible support for a *Rhynchonkos-*Gymnophiona relationship. In contrast to previous descriptions, the vomers are neither sutured nor closely integrated with the parasphenoid in *Rhynchonkos*, *Aletrimyti*, and *Dvellecanus*.

Passage of the metotic foramen through the exoccipital, rather than between the exoccipital and opisthotic, has been proposed as a characteristic uniting *Rhynchonkos* and Gymnophiona [8,9]. However, the metotic foramen in all three taxa studied here passes directly through the suture between distinct opisthotic and exoccipital bones.

The presence of an expanded ossification of the dorsum sellae (the ‘pleurosphenoid’) forming an ossified wall between the sphenethmoid and otic capsule has also been noted as a possible synapomorphy of *Rhynchonkos* and Gymnophiona. The ‘pleurosphenoid’ does not reach the sphenethmoid at all in *Rhynchonkos*, similar to the condition seen in the brachystelechid *Carrolla craddocki* [16] and the recumbirostran *Huskerpeton englehorni* [18]. The ‘pleurosphenoid’ does meet the sphenethmoid, forming a complete wall of the braincase in *Aletrimyti* and *Dvellecanus*, as in *Pelodosotis elongatum* [19], *Pantylus cordatus* [31], and *Nannaroter mckinziei* [17]. *Contra* Carroll & Currie [8], we find that the ‘pleurosphenoid’ is not particularly close to the condition in Gymnophiona; in gymnophionans, the pleurosphenoid is narrowest ventrally with a dorsal expansion that forms a robust dorsal bridge between the otic capsule and the dorsal trabecula of the sphenethmoid region, whereas in all three ‘microsaur’ taxa described here, the ‘pleurosphenoid’ is widest ventrally, and is widely separate from both otic capsule and sphenethmoid dorsally. Moreover, the antotic fenestra in gymnophionans opens laterally, whereas the antotic fenestra in all three ‘microsaur’ taxa here opens anteriorly, as in the brachystelechid *Carrolla craddocki* [16].

Previous treatments of *Rhynchonkos* have claimed presence of an incipiently dual occipital condyle, approaching the dual occipital condyle of gymnophionans [8,19]. Our study of this material does not find such an arrangement in any of the three taxa attributed to *Rhynchonkos*. Instead, we find that the occipital condyle is broad and straplike, with the standard condyle-cotyle arrangement seen in other recumbirostran ‘microsaurs’ [19]. The basioccipital participates prominently in the occipital surface, accepting a broad odontoid process of the atlas, *contra* the condition seen in Gymnophiona.

Carroll & Currie [8] claim that organization of the teeth of the coronoid into single rows also unites *Rhynchonkos* with Gymnophiona to the exclusion of all other Paleozoic tetrapods. A single row of small teeth is present on the vomer, palatine, and ectopterygoid in all three taxa studied here, but a similar row of teeth is not seen in the lower jaws of any of these taxa. In fact, the coronoid is reduced to a thin bony strut with no clear tooth row in *Aletrimyti* and is restricted far posteriorly in *Rhynchonkos* and *Dvellecanus*. This condition was primarily described from a partial isolated lower jaw that may be recumbirostran [8], but definitively cannot be attributed to any of the three taxa described here. A more robust coronoid with a clear tooth row is present in *Huskerpeton englehorni* [18] and it is possible that the lower jaw comes from a similar animal.

The presence of a strong retroarticular process of the lower jaw has also been noted as a possible synapomorphy of *Rhynchonkos* and Gymnophiona. The retroarticular process is strongly developed in *Aletrimyti*, but it is only weakly-developed in *Rhynchonkos* and is absent in *Dvellecanus*.

Although it is ultimately possible that one or more of these characters may provide support for a relationship between recumbirostran ‘microsaurs’ and gymnophionans, it is likely that the strong support for a relationship between *Rhynchonkos* and gymnophionans resulted in most cases from the chimaeric nature of *Rhynchonkos* as previously described. In other cases, this may reflect difficulties of studying these specimens without micro-CT. Furthermore, there is little consistency in the distribution of gymnophionan characteristics among the three species previously attributed to *Rhynchonkos*; no one taxon is considerably more gymnophionan-like than any other. These characteristics (specifically the retroarticular process and expanded ossification of the ‘pleurosphenoid’ contacting the sphenethmoid) may represent fossorial adaptations rather than homologies shared with caecilians, in which case the origins of caecilians must be sought elsewhere.
